# Oleaceae plants: a source of metabolites with atheroprotective potential

**DOI:** 10.3389/fphar.2025.1721433

**Published:** 2026-02-11

**Authors:** Agnieszka Filipek, Edyta Czepielewska

**Affiliations:** 1 Department of Pharmaceutical Biology, Medical University of Warsaw, Warsaw, Poland; 2 School of Health and Medical Sciences, Vizja University, Warsaw, Poland

**Keywords:** active metabolites., atherosclerosis, hydroxytyrosol, Oleaceae, olive oil

## Abstract

Atherosclerosis is a leading cause of cardiovascular morbidity and mortality, driven by chronic inflammation, oxidative stress, lipid imbalance, and plaque instability. Bioactive metabolites found in plants of the Oleaceae family, particularly olives (*Olea europaea* L.), have emerged as promising modulators of these processes. This narrative review highlights several key phytochemicals, such as oleacein and oleuropein (secoiridoids), hydroxytyrosol and oleocanthal (phenylethanoids), acteoside and syringin (phenylpropanoids), oleanolic and ursolic acids (triterpenoids), phillygenin (lignans), caffeic and chlorogenic acids (phenolic acids). These metabolites act by activating the Nrf2/HO-1 antioxidant defence pathway, inhibiting NF-κB-driven inflammation, regulating cholesterol transport (ABCA1/ABCG1 and CD36), protecting the endothelium, and stabilising atherosclerotic plaque. Preclinical studies consistently demonstrate strong anti-atherosclerotic activity, while clinical evidence mainly supports the consumption of polyphenol-rich extra virgin olive oil, which has been linked to improved endothelial function, reduced oxidative and inflammatory markers, and a lower risk of cardiovascular disease. However, robust trials of purified compounds and non-olive Oleaceae species are lacking. Oleaceae-derived phytochemicals offer multi-targeted cardioprotective potential, but their translation into clinical applications remains limited.

## Graphical Abstract

**Figure:**
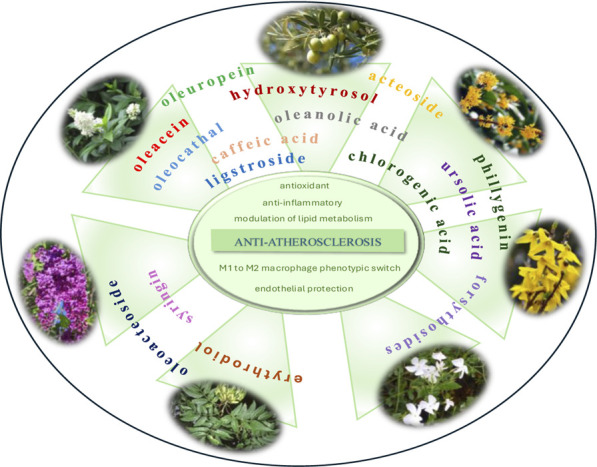

## 1 Introduction

Atherosclerosis, a chronic inflammatory disease affecting arterial walls, stands as a primary contributor to global morbidity and mortality, frequently culminating in myocardial infarction or ischemic stroke (Bjorkegren and Lusis, 2022). The condition is hallmarked by the accumulation of lipids, immune cells, and extracellular matrix components within the intimal layer of arteries, leading to the formation of atherosclerotic plaques that progressively narrow the arterial lumen (Chan and Ramji, 2022). The pathogenesis of atherosclerosis is a complex interplay of various cellular and molecular events, encompassing endothelial dysfunction, lipoprotein oxidation, inflammatory cell recruitment, and vascular smooth muscle cell proliferation (Yu et al., 2024). Atherosclerotic plaques are composed of a fibrous cap, which is mostly collagen, and a lipid core, which contains fats, cholesterol, white blood cells, and proteins (Melamed and Goldhaber, 2014). This structure of atherosclerotic plaques makes them susceptible to rupture, which leads to the formation of blood clots and acute cardiovascular incidents (Chung, 2016).

The evolution of atherosclerotic changes begins with damage to the endothelium, the inner lining of blood vessels. This damage can be caused by various factors, such as hypertension, smoking, high cholesterol, diabetes, or chronic inflammation. Endothelial damage and lipid deposition trigger an inflammatory response. Immune cells (monocytes and macrophages) migrate to the vessel wall, where they engulf LDL, transforming into foam cells. Foam cells, along with other components (e.g., smooth muscle cells, collagen), form the core of the atherosclerotic plaque. This core becomes covered with a fibrous cap, creating a relatively stable lesion. However, as deposits accumulate, the atherosclerotic plaque gradually enlarges, and the progressive inflammatory process in the vessel wall is associated with the development of unstable atherosclerotic lesions. Unstable atherosclerotic plaques are characterized by increased vascularization, a thinner, rupture-prone fibrous cap, and an increased number of inflammatory cells. The lipid core of the plaque becomes voluminous and rich in liquid cholesterol esters. Abnormal and expanding vessels are the main source of hemorrhage into the plaque and its periphery, which ultimately leads to its rupture, resulting in clots that completely block the vessels. The clot can break off and migrate to another vessel, causing an embolism. Insufficient blood flow to organs can lead to ischemia and, consequently, heart attack, stroke, intermittent claudication (in the lower limbs), or other complications, depending on the location of the lesion (Jebari-Benslaiman et al., 2022; Sachdeva et al., 2023).

**FIGURE 1:**
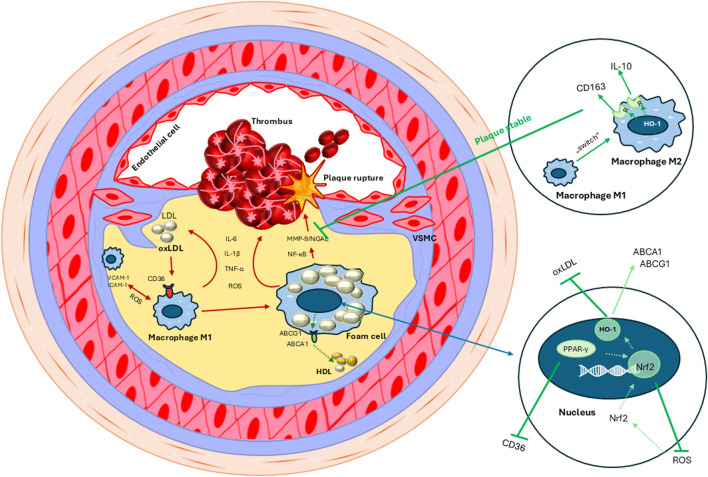
Activation of VCAM-1 and ICAM-1 by ROS (reactive oxygen species) stimulates the influx and adhesion of M1 (proinflammatory) macrophages to the vessel wall. Production of proinflammatory markers (IL-1β, IL-6, TNF-α) in macrophages stimulates expression of the CD36 receptor and increases the accumulation of oxidized lipids (oxLDL) in cells. As a result of unrestricted cholesterol uptake, macrophages transform into atherogenic foam cells, which form atherosclerotic plaques by producing proinflammatory and oxidative factors. Over time, the atherosclerotic plaque becomes unstable and ruptures, leading to microhemorrhages and thrombus formation (red arrows). Activation of PPAR-γ and oxidative stress induced by ROS allows Nrf2 to enter the cell nucleus and activate the expression of genes encoding antioxidant and detoxifying enzymes, such as HO-1. This enzyme can activate reverse cholesterol transport from macrophages *via* the ABCA1 and ABCG1 transporters (green arrows). The M1 to M2 (anti-inflammatory) switch in macrophages initiates the production of anti-inflammatory factors (CD163, IL-10, HO-1) that inhibit MMP-9/NGAL expression, thereby stabilizing the atherosclerotic plaque (green arrows).

Ultimately, the consequence of atherosclerosis is chronic inflammation. Inflammation plays a pivotal role in all stages of atherogenesis, with inflammatory cells, such as macrophages and T-lymphocytes, actively participating in plaque formation and destabilization (Koga et al., 2013; Spagnoli et al., 2007). Understanding the molecular mechanisms underpinning atherosclerosis is crucial for identifying potential therapeutic targets aimed at preventing or mitigating the disease (Figure 1).

The Oleaceae family, encompassing plants like olive (*Olea europaea* L.), privet (*Ligustrum vulgare* L.), ash (*Fraxinus excelsior* L.), jasmine (*Jasminum sp.- Dwarf White Jasmine*), forsythia (*Forsythia* × *intermedia* Zabel), sweet olive (*Osmanthus fragrans* Lour.), and lilac (*Syringa vulgaris* L.), has garnered significant attention in recent years due to its rich reservoir of bioactive metabolites with promising therapeutic potential, particularly in the context of cardiovascular health (Ryan et al., 2002). Considering the need to search for new preventive and therapeutic strategies for atherosclerosis, the Oleaceae plant family seems to be a potential source of phytochemicals exhibiting a number of pharmacological activities relevant to both early and late stages of atherosclerosis development. These activities include antioxidant, anti-inflammatory, and lipid-modulating effects, suggesting a multi-pronged approach to combating this complex disease. The presence of phenolic compounds such as oleuropein, oleacein, oleocanthal, and hydroxytyrosol, as well as triterpenoids like oleanolic acid, contributes to the atheroprotective potential of Oleaceae plants (Diamantakos et al., 2021; Pollier and Goossens, 2012).

Many medications are used to treat atherosclerosis, primarily statins, which lower blood cholesterol levels, and antiplatelet drugs with anticoagulant properties. Unfortunately, synthetic drugs cause numerous side effects. Biocomponents appear to be a good alternative. Therefore, this paper aims to delve into the metabolites found in Oleaceae plants that demonstrate the ability to inhibit early and late atherosclerotic changes, elucidating the underlying mechanisms of action and highlighting their potential for future applications in cardiovascular disease prevention and treatment.

## 2 Methods

This article is designed as a narrative review, with the aim to provide an updated overview of the anti-atherosclerotic potential of metabolites derived from plants of the Oleaceae family. The review focuses on their chemical diversity, molecular targets, preclinical evidence, and clinical implications.

### 2.1 Literature search strategy

Relevant literature was identified by searching PubMed/MEDLINE, Embase, Scopus, Web of Science, and Cochrane CENTRAL databases up to September 2025. The search combined terms related to atherosclerosis (“atherosclerosis”, “endothelial dysfunction”, “macrophage”, “inflammation”, “lipid oxidation”, “foam cell”, “cholesterol efflux”, “atherosclerotic plaque”) with those related to Oleaceae plants and their bioactive metabolites (“Oleaceae”, “acteoside”, “caffeic acid”, “chlorogenic acid”, “erythrodiol”, “forsythoside”, “hydroxytyrosol”, “ligstroside”, “oleacein”, “oleanolic acid”, “oleoacteoside”, “oleocanthal”, “oleuropein”, “phillygenin”, “syringin”, “ursolic acid”, “olive oil”). The search strategy, including examples of search strings, is presented in Table 1. All of the listed keywords were combined in this manner.

**TABLE 1:** Literature search strategy.

Identification	Records were identified through database searches using keyword combinations related to atherosclerosis and Oleaceae phytochemicals (e.g., “atherosclerosis” and “Oleaceae”, “atherosclerosis” and “acteoside”).
Screening	Duplicate records from multiple databases have been removed
	Titles and abstracts were screened for relevance to Oleaceae phytochemicals, mechanisms linked to atherosclerosis and identifiable compound composition
	Full articles were evaluated for the presence of mechanistic, preclinical, or clinical data; appropriate compound identification; and relevance to oxidative stress, inflammation, lipid transport, endothelial function, or plaque stability. Efforts were made to find articles presenting metabolites from the Oleaceae family in the broadest possible anti-atherosclerotic context
Included	Studies contributing mechanistic, preclinical and clinical insights grouped thematically

### 2.2 Inclusion and exclusion criteria

Studies were included if they met the following criteria:

- Evaluated individual bioactive metabolites naturally found in Oleaceae species or *Oleaceae*-derived substances (e.g., olive oil),
- Investigated anti-atherosclerotic, antioxidant, anti-inflammatory, lipid-modulating, endothelial-protective or plaque-stabilizing effects,
- Were experimental (*in vitro*, *in vivo*) or clinical studies,
- Clearly identified the metabolites under investigation,
- Were published in English and available in full text.

We excluded studies that:

- Investigated plant extracts not specific to the Oleaceae family,
- Lacked chemical characteristics of the tested substances,
- Were reviews without original data (unless used to provide mechanistic context),
- Focused solely on agricultural, botanical, or food technology aspects without biomedical relevance.

### 2.3 Data synthesis

Data were synthesized narratively due to heterogeneity in metabolite purity, dosing, experimental models, and outcomes. Evidence was stratified into three predefined categories:

- Clinical trials–prioritized as the most relevant for translation;
- *In vivo* studies–with ApoE^−/−^, LDL-R^−/−^ and diet-induced models emphasized;
- *In vitro* studies–used to clarify molecular pathways.

### 2.4 Plant materials, metabolites, commercial products

The quality of plant material, metabolites, and commercial products (olive oil) in the studies included in this review was assessed prior to their use in experiments. The plant material such as *Ligustrum vulgare* L., (Oleaceae, *L. vulgare folium*) (Filipek et al., 2017; Filipek and Gierlikowska, 2021; Kiss, Mank, Melzig, 2008; Parzonko et al., 2013; Peyrot des Gachons et al., 2024), *Olea europaea* L. (Oleaceae, *O. europaea folium*) (Silvestrini et al., 2023; Christodoulou et al., 2024), *Syringa vulgaris* L., Oleaceae, *S. vulgaris cortex* (Filipek et al., 2019), *Forsythia viridissima* Lindl., (Oleaceae, *Forsythia viridissima fructus*) (Lee et al., 2010), *Forsythia x intermedia* Zabel (Oleaceae, *Forsythia x intermedia flos*) (Michalak et al., 2018), *Forsythia suspensa* L. (Oleaceae, *Forsythia suspensa fructus et folium*) (Sung et al., 2016; Guo et al., 2022a), *Osmanthus fragrans* Loureiro (Oleaceae, *O. fragrans flos*) (Hung et al., 2012; Song et al., 2018), *Fraxinus excelsior* L. (Oleaceae, *F. excelsior folium*) (Kołtun-Jasion et al., 2023) as well as *Cistanche tubulosa* (Schenk) Wight (Orobanchaceae) (Muhtar et al., 2025), *Cistanche deserticola* Ma. (Orobanchaceae) (Jia et al., 2023), *Tinospora crispa* L. (Menispermaceae, *T. crispa caulis*) (Arshad, et al., 2024), *Rhaponticum carthamoides* Willd. (Asteraceae, *R. carthamoides herba*) (Nan et al., 2024) was identified on the basis of anatomical and morphological characters using the microscopic method described, for example, in the monographs of the Pharmacopeia or in Flora Europaea. The collection of plant material was described in detail and its voucher specimen with the appropriate number was deposited in the appropriate Plant Collection.

The plant material from which the metabolites were isolated was also extra virgin olive oil. The olive oil samples were also thoroughly described (country, region, harvest season, variety, *etc.*) (Christodoulou et al., 2024; Gutierrez-Miranda et al., 2020; Katsa et al., 2024; Montoya et al., 2018; Montoya et al., 2019; Montoya et al., 2021; Montoya et al., 2023; Qosa et al., 2015; Salsano et al., 2022; Wang et al., 2017).

The isolation and identity confirmation of plant metabolites were performed using chemical analyses such as FCPE, FCPC, LOQ, LOD, TLC, LC-HRMS, HPLC/MS/MS, QTOF-MS/MS and NMR (Supplementary Material). Metabolites with a purity of at least 95% were used in the studies (Arshad et al., 2024; Christodoulou et al., 2024; Diamantakos et al., 2021; Filipek et al., 2015; Filipek et al., 2019; Filipek and Gierlikowska, 2021; Filipek et al., 2025; Guo et al., 2022b; Gutierrez-Miranda et al., 2020; Hung et al., 2012; Jia et al., 2023; Kiss, Mank, Melzig, 2008; Kołtun-Jasion et al., 2023; Lee et al., 2010; Michalak et al., 2018; Montoya et al., 2018; Montoya et al., 2019; Montoya et al., 2021; Montoya et al., 2023; Muhtar et al., 2025; Nan et al., 2024; Qosa et al., 2015; Song et al., 2018).

However, most metabolites were obtained from commercial sources. A detailed summary can be found in the Supplementary Material.

Extracts of extra virgin olive oil (EVOO) and olive mill wastewater (OMWW) were evaluated using HPLC and LC-MS-MS (Hara et al., 2023; Cuffaro et al., 2024). Identification of phenolic compounds in the olive oil was by UHPLC-HRMS (Geana et al., 2023). Some studies were based on semi-quantitative surveys (Donat-Vargas et al., 2022; Donat-Vargas et al., 2023). In two works, oleacein was synthesized from other compounds (e.g., oleuropein) using ESI-MS and NMR methods (Rosillo et al., 2024; Shimamoto et al., 2023).

Other studies used dietary supplements produced specifically for the study by commercial companies, such as Aquilea Colesterol® Novel Nutraceutic (Domenech et al., 2019), capsules containing a known amount of hydroxytyrosol (Quirós-Fernández et al., 2019) and oleuropein (Romero et al., 2016).

## 3 Bioactive metabolites in plants belonging to the Oleaceae family

Plants from the Oleaceae family are characterized by a variety of bioactive metabolites, including phenylpropanoids, phenylethanoids, secoiridoids and triterpenoids contributing in a unique way to their potential therapeutic properties.

### 3.1 Phenylpropanoids

#### 3.1.1 Acteoside

Acteoside (also known as verbascoside, Table 2) is a phenylpropanoid glycoside widely distributed among higher plants, particularly in the Oleaceae family. It is found in olives and olive oil, particularly concentrated in the fruit and leaves (*O. europaea* L. Oleaceae, *O. europaea fructus et folium*), and in olive mill effluents as well as *O. fragrans flower* (*O. fragrans* Lour., Oleaceae) (Ryan et al., 2002; Li et al., 2023). It has been demonstrated to exhibit strong free radical-scavenging capacity, decreases ROS and lipid peroxidation, while restoring endogenous antioxidant enzymes (SOD, CAT, GPx) (Yang et al., 2023). Acteoside upregulates cholesterol efflux by ABCA1 transporter, contributing to the reduction of foam cell formation (Jia et al., 2023). Moreover, acteoside inhibits key pro-inflammatory NF-κB signaling pathway and lowers production of cytokines such as IL-1β, IL-6 and TNF-α (Muhtar et al., 2025).

**TABLE 2:** Bioactive metabolites from the Oleaceae family.

No	Metabolite	Synonims	Molecular weight (g/mol)	Chemical name	Chemical structure
1.	Hydroxytyrosol	2-(3,4-dihydroxyphenyl)ethanol	154.16	4-(2-hydroxyethyl)benzene-1,2-diol	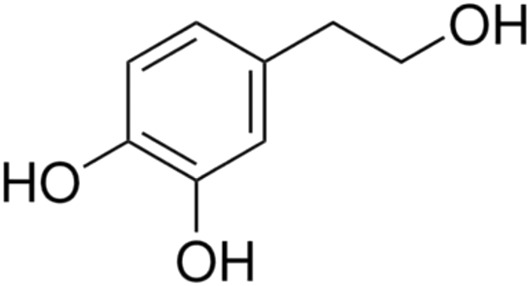
2.	Oleuropein	Oleoeuropein Oleoeuropeine	540.5	2-(3,4-dihydroxyphenyl)ethyl [2S-(2alpha,3E,4beta)]-3-ethylidene-2-(beta-D-glucopyranosyloxy)-3,4-dihydro-5-(methoxycarbonyl)-2H-pyran-4-acetate	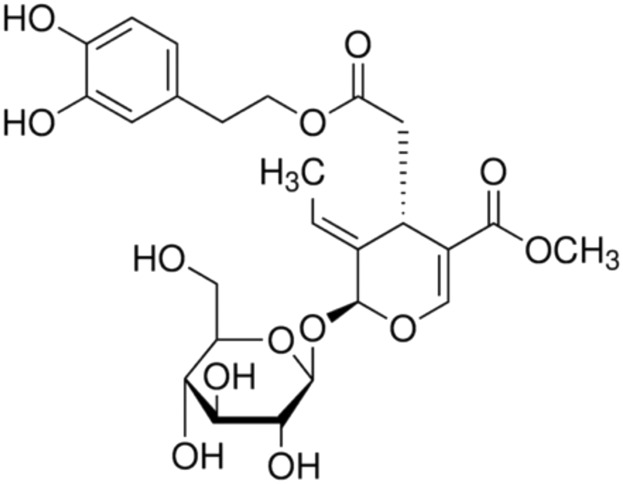
3.	Oleacein	3,4-DHPEA-EDA	320.3	2-(3,4-dihydroxyphenyl)ethyl (Z)-4-formyl-3-(2-oxoethyl)hex-4-enoate or (3S,4E)-4-Formyl-3-(2-oxoethyl)-4-hexenoic acid 2-(3,4-Dihydroxyphenyl)ethyl ester	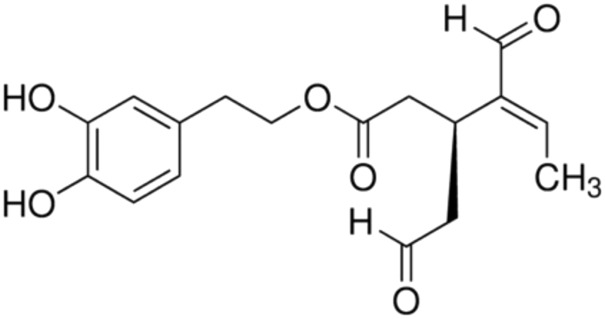
4.	Oleocanthal	(−)-Oleocanthal Deacetoxy ligstroside aglycon	304.34	2-(4-hydroxyphenyl)ethyl (3S)-4-formyl-3-(2-oxoethyl)hex-4-enoate	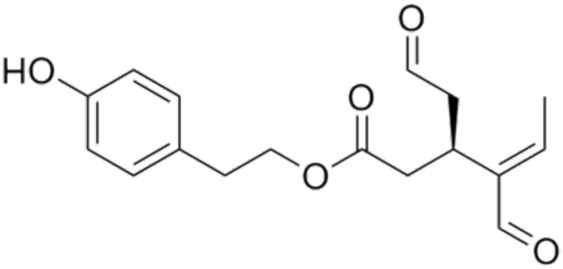
5.	Oleanolic acid	Oleanic acid Caryophyllin Astrantiagenin C Oleanol	456.7	3(β)-3-hydroxyolean-12-en-28-oic acid	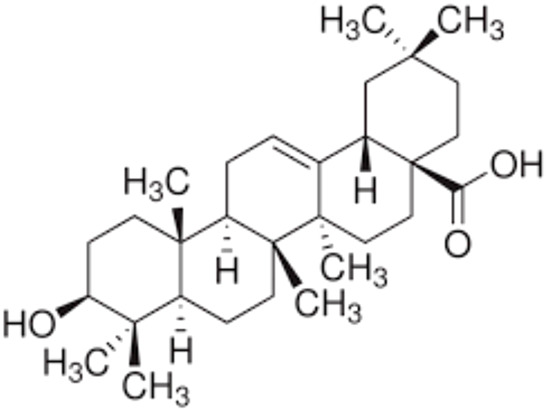
6.	Ursolic acid	Prunol Malol Urson	456.7	3(β)-3-hydroxyurs-12-en-28-oic acid	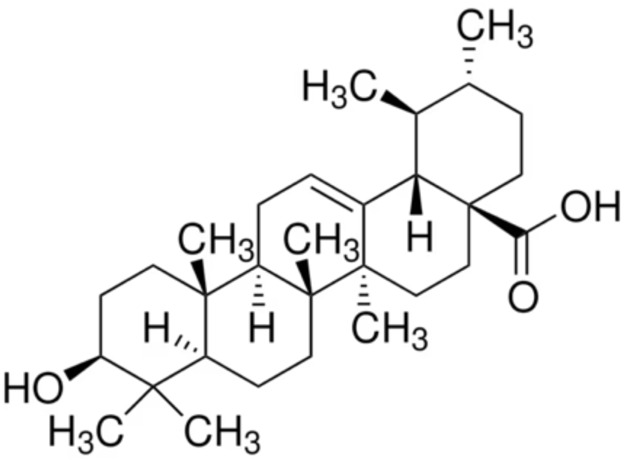
7.	Erythrodiol	3beta-erythrodiol	442.7	Olean-12-ene-3beta,28-diol	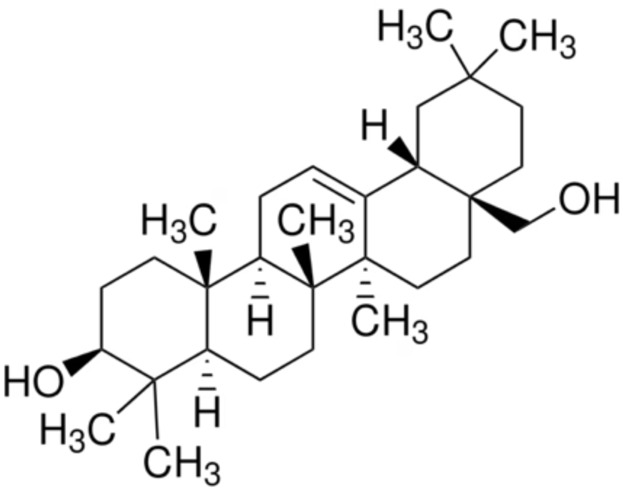
8.	Forsythoside A	Forsythiaside	624.6	[(2*R*,3*S*,4*R*,5*R*,6*R*)-6-[2-(3,4-dihydroxyphenyl)ethoxy]-4,5-dihydroxy-2-[[(2*R*,3*R*,4*R*,5*R*,6*S*)-3,4,5-trihydroxy-6-methyloxan-2-yl]oxymethyl]oxan-3-yl] (*E*)-3-(3,4-dihydroxyphenyl)prop-2-enoate	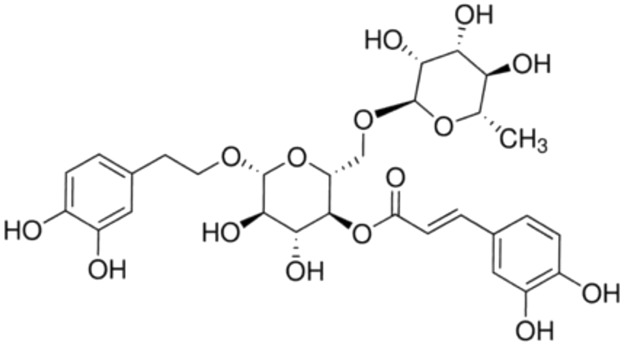
​	Forsythoside B	n/d	756.7	[(2*R*,3*R*,4*R*,5*R*,6*R*)-2-[[(2*R*,3*R*,4*R*)-3,4-dihydroxy-4-(hydroxymethyl)oxolan-2-yl]oxymethyl]-6-[2-(3,4-dihydroxyphenyl)ethoxy]-5-hydroxy-4-[(2*S*,3*R*,4*R*,5*R*,6*S*)-3,4,5-trihydroxy-6-methyloxan-2-yl]oxyoxan-3-yl] (*E*)-3-(3,4-dihydroxyphenyl)prop-2-enoate	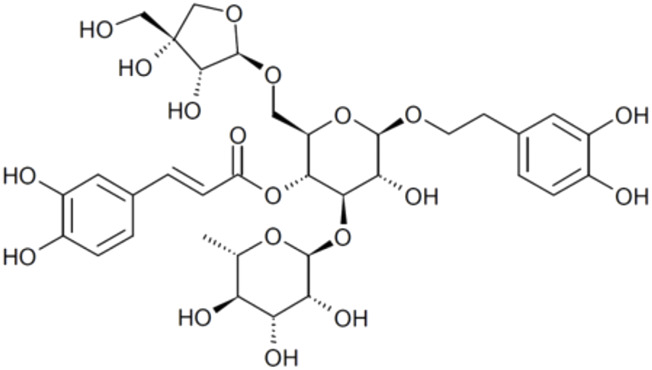
9.	Syringin	Eleutheroside B Ligustrid	372.4	4-[(1E)-3-hydroxyprop-1-en-1-yl]-2,6-dimethoxyphenyl β-D-glucopyranoside	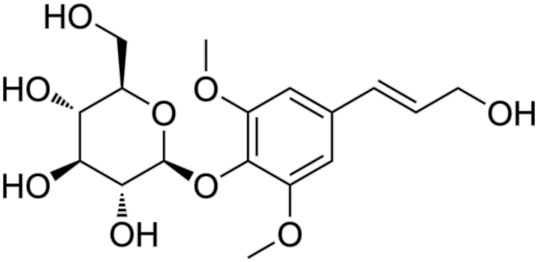
9.	Phillygenin	Epipinoresinol methyl ether (+)-Phillygenin	372.4	4-[(1S,3aR,4R,6aR)-4-(3,4-Dimethoxyphenyl)tetrahydro-1H,3H-furo[3,4-c]furan-1-yl]-2-methoxyphenol	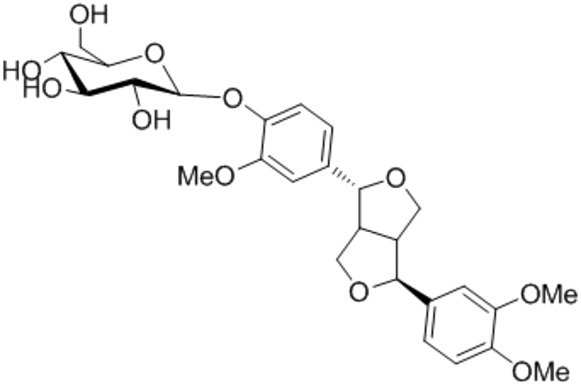
10.	Acteoside	Verbascoside Kusaginin	624.6	β-(3,4-dihydroxyphenylethyl)-O-α-L-rhamnopyr-anosyl-(1→3)-β-D-(4-O-caffeoyl)-glucopyranoside	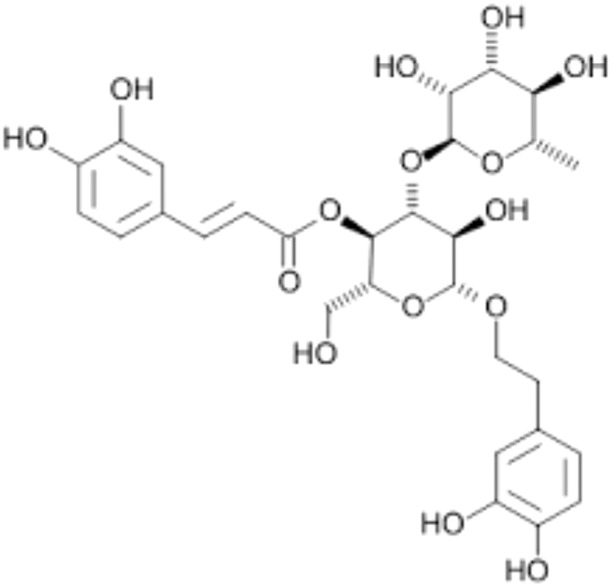
11.	Ligstroside	Ligustroside	524.5	methyl ester of 3,4-dihydro-2H-pyran-5-carboxylic acid	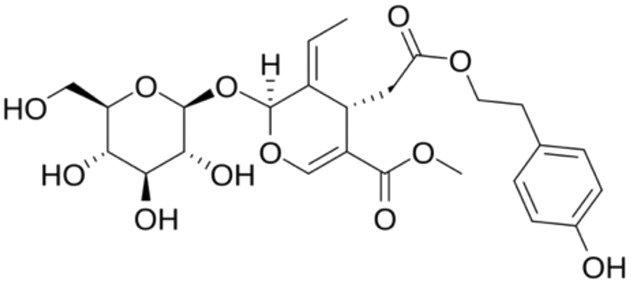
12.	Oleoacteoside	n/d	1,010.9	β-(3,4-dihydroxyphenylethyl)-O-α-L-rhamnopyr-anosyl-(1→3)-β-D-(4-O-caffeoyl)-glucopyranoside-O-[(2-ropen-1-yloxy)carbonyl]	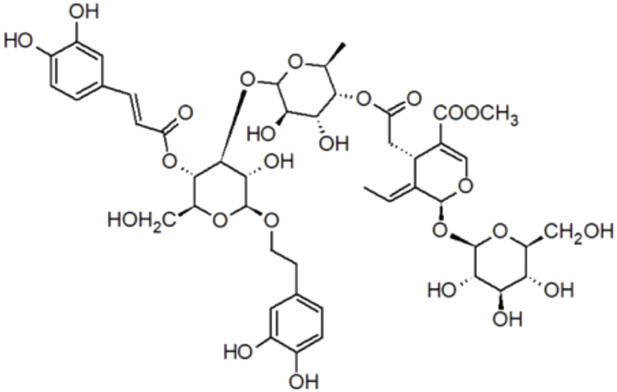
13.	Chlorogenic acid	Heriguard Caffeoyl quinic acid (+)-Chlorogenic acid	354.31	3-(3,4-Dihydroxycinnamoyl)quinic acid	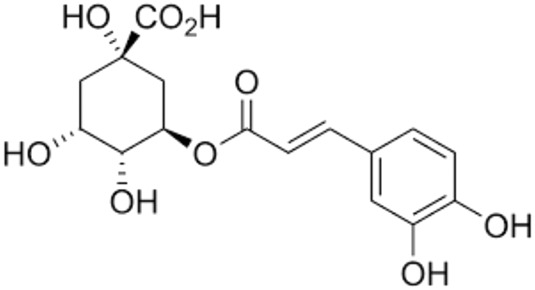
14.	Caffeic acid	cis-Caffeic acid trans-caffeic acid	180.16	3,4-Dihydroxycinnamic acid	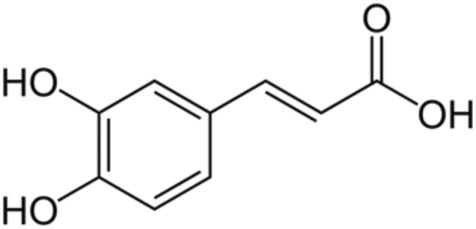

#### 3.1.2 Syringin

Syringin (Table 2), a phenylpropanoid glycoside found in various parts of the lilac plant (*S. vulgaris* L., Oleaceae) as well as bark of ash (*F. excelsior* L*.,* Oleaceae, *F. excelsior cortex*) (Kołtun-Jasion et al., 2023; Liu et al., 2025), has been shown to exhibit antioxidant and anti-inflammatory activities, which could be beneficial in preventing or slowing down the progression of atherosclerosis (Zhao et al., 2023). By alleviating the oxidative-redox imbalance leading to oxidative stress, syringin can protect LDL cholesterol from oxidation (Wang et al., 2020; Filipek et al., 2025). Additionally, syringin has been reported to inhibit the production of pro-inflammatory cytokines, such as TNF-α and IL-1β, which play a key role in the inflammatory processes that contribute to atherosclerosis (Arshad et al., 2024). Moreover, it can eliminate the dysfunction of vascular endothelial cells (Nan et al., 2024).

### 3.2 Secoirydoids

#### 3.2.1 Oleuropein

Oleuropein (Table 2), a secoiridoid glycoside abundantly found in olive leaves and fruits (*O. europaea* L. Oleaceae, *O. europaea fructus et folium*) and of different species of Oleaceae family (Khalil et al., 2024), has been extensively studied for its diverse pharmacological activities, including its ability to prevent atherosclerosis. Oleuropein, along with its aglycone form, exhibits potent antioxidant and anti-inflammatory properties. Studies have demonstrated that oleuropein can activate sirtuin 1 (SIRT1) gene expression (Hu et al., 2025), which plays a crucial role in preventing endothelial dysfunction, and suppresses the expression of adhesion molecules (ICAM, VCAM) on endothelial cells, thereby preventing the recruitment of inflammatory cells to the arterial wall. Moreover, oleuropein can inhibit the activation of NF-κB, a major transcription factor that regulates the expression of pro-inflammatory genes, thereby reducing the production of inflammatory cytokines and chemokines (Silvestrini et al., 2023).

#### 3.2.2 Oleacein

Oleacein (Table 2) is a secoiridoid present in olive fruits and leaves. In terms of chemical structure, oleacein is a derivative of oleuropein glycoside and its structural changes are strongly affected by the extraction conditions used during the process of pressing oil (Peralta et al., 2025; Cuffaro et al., 2024). Additionally, oleacein can be isolated in significant amounts from leaves of *L. vulgare* L. (Oleaceae, *L. vulgare folium*) (Peyrot des Gachons et al., 2024). Oleacein has shown significant anti-inflammatory and antioxidant properties. Its molecular structure allows for efficient scavenging of free radicals, protecting lipids and other biomolecules from oxidative damage. Additionally, oleacein has been shown to inhibit the production of pro-inflammatory cytokines, such as TNF-α and IL-1β, which play a crucial role in the progression of atherosclerosis (Filipek and Gierlikowska, 2021). Other studies have shown that oleacein increases the expression of the CD163 receptor and IL10, reducing microhemorrhages, which reduces inflammation, e.g., within the atherosclerotic plaque, preventing its destabilization (Filipek et al., 2015). Moreover, oleacein has been shown to interfere with the activation of the NLRP3 inflammasome (Gutierrez-Miranda et al., 2020), a multi-protein complex that triggers the release of pro-inflammatory cytokines, thereby reducing inflammation in the arterial wall. Furthermore, oleacein can modulate the activity of enzymes involved in lipid metabolism, potentially leading to a reduction in LDL cholesterol levels and an increase in HDL cholesterol levels (Ruiz-García et al., 2023).

#### 3.2.3 Ligstroside

Ligstroside (Table 2) is one of the main secoiridoids found in extra virgin olive oil. In terms of chemical structure, it differs from oleuropein by a single hydroxyl group. It is known that during olive oil pressing, ligstroside can undergo hydrolysis into other bioactive metabolites, such as tyrosol (Efantakis et al., 2015). It has been found that ligstroside, like other secoiridoids, exhibits biological activities relevant to atherosclerosis prevention, i.e., strong antioxidant and anti-inflammatory properties (Castejon et al., 2022).

### 3.3 Phenylethanoids

#### 3.3.1 Hydroxytyrosol

Hydroxytyrosol (Table 2), a phenylethanoid prominently found in olive oil and olive leaves (*O. europaea* L. Oleaceae, *O. europaea folium*) (Elhrech et al., 2024), stands out as a potent antioxidant with a diverse array of biological activities relevant to atherosclerosis prevention. Its ability to scavenge free radicals, inhibit LDL oxidation, and reduce inflammation positions it as a promising candidate for mitigating early atherosclerotic changes. Moreover, hydroxytyrosol has demonstrated the capacity to modulate endothelial function, promoting vasodilation and preventing the adhesion of immune cells to the arterial wall, thereby further hindering the initiation and progression of atherosclerosis. At the molecular level, hydroxytyrosol has been shown to activate the Nrf2 pathway, a key regulator of cellular antioxidant defenses, leading to the upregulation of antioxidant enzymes such as superoxide dismutase and catalase (Wen et al., 2024). Additionally, hydroxytyrosol can inhibit the activity of pro-inflammatory enzymes such as cyclooxygenase-2 (COX-2) and inducible nitric oxide synthase (iNOS), thereby reducing the production of inflammatory mediators. Hydroxytyrosol also exhibits antiplatelet activity (Batarfi et al., 2024). The European Food Safety Authority has acknowledged the positive effects of olive oil polyphenols, including hydroxytyrosol, in protecting blood lipids from oxidative stress (Salsano et al., 2022).

#### 3.3.2 Oleocanthal

Oleocanthal (Table 2), a unique phenylethanoid found in extra virgin olive oil, has gained attention for its strong anti-inflammatory properties, particularly its ability to inhibit cyclooxygenase enzymes (COX), similar to non-steroidal anti-inflammatory drugs. Oleocanthal exerted even better capability to reduce inflammation than ibuprofen, at the same concentration (Montoya et al., 2021). By inhibiting COX-1 and COX-2, oleocanthal can reduce the production of pro-inflammatory prostaglandins, thereby mitigating inflammation in the arterial wall and potentially slowing down the progression of atherosclerosis. Oleocanthal has been shown to suppress the activation of NF-κB, a key transcription factor that regulates the expression of pro-inflammatory genes, thereby reducing the production of inflammatory mediators (Carpi et al., 2019).

#### 3.3.3 Oleoacteoside

Oleoacteoside (Table 2) is a natural phenylethanoid found in some plants, particularly within the Oleaceae family. It is primarily found in plants like *Syringa reticulata subsp. amurensis (Rupr*.) P.S.Green and M.C.Chang (Japanese tree lilac), *Syringa josikaea* J. Jacq. *ex* Rchb (Hungarian lilac) and *S. vulgaris* L. (common lilac) as well as *F. excelsior* L*.* (common ash) (Dudek et al., 2017; Kołtun-Jasion et al., 2023). Oleoacteoside has strong anti-inflammatory properties by inhibiting IL-1β, IL-6 and TNF-α. Furthermore, scientists suggest, that with acteoside these two metabolites work synergistically to achieve the ultimate anti-inflammatory effect (Kołtun-Jasion et al., 2023).

#### 3.3.4 Forsythosides

Forsythosides (Table 2) belong to the phenylethanoid glycosides and are found mainly in the fruits of *Forsythia suspensa* (Thunb.) Vahl (Oleaceae, *Forsythia suspensa fructus*) and *Jasminum mesnyi* Hance (Oleaceae, *J. mesnyi fructus*) Based on their functional groups, these metabolites are classified into several groups, from A to K (Yang et al., 2022). Recent studies have shown that forsythosides have a protective effect on the cardiovascular system. Following intraperitoneal injection of forsythoside A to mice with heart failure, the production of pro-inflammatory cytokines, such as TNF-α, IL-6, and IL-1β, was significantly reduced, and the expression of the transcription factor NF-κB was inhibited (Fu et al., 2021). Also forsythoside B can suppress the expression of pro-inflammatory cytokines, as well as enzymes (iNOS, COX-2) by inhibiting NF-κB signaling pathway (Wang et al., 2025; Xia et al., 2022). Both metabolites activate the Nrf2 pathway, enhancing cellular antioxidant capacity and reducing oxidative stress (Li et al., 2025; Chen et al., 2023a).

### 3.4 Triterpenoids

#### 3.4.1 Oleanolic acid

Oleanolic acid (Table 2) is a pentacyclic triterpene found widely in the plant kingdom, common in the Oleaceae family, especially in the fruit, leaves and bark of the olive tree (*O. europaea* L. Oleaceae, *O. europaea folium, fructus et cortex*). Therefore, it is a natural component of extra virgin olive oil (Pollier and Goossens, 2012; Guinda et al., 2010). Oleanolic acid exhibits potent anti-inflammatory and antioxidant properties. Scientists have also confirmed its anti-atherosclerotic and antihypertensive bioactivity (Fernandez-Aparicio et al., 2019). Oleanolic acid was confirmed to effectively alleviate HUVEC damage by inhibiting ROS production and LOX-1 expression, as well as increasing Nrf2/HO-1 levels (Jiang et al., 2015). Another study showed that oleanolic acid inhibits NF-κB signaling, resulting in decreased production of inflammatory mediators such as PGE2, TNF-α and IL-6 (Hwang et al., 2014). In hyperlipidemic mice, oleanolic acid increased macrophage cholesterol efflux by ABCA1 and ABCG1 transporters (Zhang et al., 2016). Moreover, it possesses endothelial protection properties *via* eNOS activation (Zhang et al., 2018).

#### 3.4.2 Erythrodiol

Erythrodiol (Table 2) is one of the pentacyclic compounds found in olive and ash leaves (*F. excelsior* L., Oleaceae*, F. excelsior folium*). This metabolite has not yet been thoroughly investigated for its anti-atherosclerotic activity. However, conducted research has shown that erythrodiol can stimulate the activity of the ABCA1 transporter and enhance cholesterol efflux from foam cells (Wang et al., 2017).

#### 3.4.3 Ursolic acid

Ursolic acid (Table 2) is a naturally occurring pentacyclic carboxylic triterpenoid found, among others, in the leaves of the *O. europaea* L. (Oleaceae, *O. europaea folium*) (Stiti and Hartmann, 2012), fruits of *Forsythia viridissima* Lindl. (Oleaceae, *Forsythia viridissima fructus)* (Lee et al., 2010) and *Ligustrum lucidum* W.T.Aiton (Dong et al., 2022). It is known for its diverse biological properties, including anti-inflammatory and antioxidant actions (Zhang et al., 2017). *In vitro* studies have shown that ursolic acid significantly reduces LOX-1 expression in the endothelium induced by LPS, at both the mRNA and protein levels. Pretreatment with ursolic acid also inhibited LPS-activated TLR4/MyD88 signaling, reduced ROS production, and inhibited NF-κB. These findings were confirmed in a model of ApoE^−/−^ mice fed an atherogenic diet, both ursolic acid (100 mg/kg/day) and simvastatin. Ursolic acid significantly reduced atherosclerotic plaque formation and reduced the area of the necrotic core (Li et al., 2018).

### 3.5 Lignans

#### 3.5.1 Phillygenin

Phillygenin (Table 2) belongs to the group of lignans, compounds that are precursors to phytoestrogens. The leaves, flowers, and fruits of various forsythia species (*Forsythia × intermedia* Zabel, Oleaceae, *Forsythia × intermedia folium, flos et fructus*) (Michalak et al., 2018) as well as flowers of *O. fragrans* Lour. (Oleaceae, *O. fragrans flos*) (Hung et al., 2012) are particularly rich in phillygenin. Zhou et al. (2021) demonstrated *in vivo* that phillygenin inhibits the production of prostaglandin E2 (PGE2) and the expression of matrix-degrading enzymes in rat chondrocytes, which may be related to its bioactivity, useful in the treatment of osteoarthritis. In turn, Liu et al. (2019) revealed the beneficial properties of phillygenin in alleviating hypertension in rats. In the context of cardiovascular disease, our recent studies have shown that phillygenin enhances reverse cholesterol transport from macrophages by activating the ABCA1 transporter. Furthermore, increased ABCA1 expression was associated with upregulation of the HO-1/Nrf2-dependent pathway (Filipek et al., 2025). This clearly demonstrates that phillygenin may be a valuable botanical drug used not only in the regression of early atherosclerotic lesions but also as an adjunct in the treatment of other inflammatory diseases.

### 3.6 Phenolic acids

#### 3.6.1 Caffeic acid

Caffeic acid (Table 2) is a representative of hydroxycinnamic acids, a subgroup of phenolic acids. Caffeic acid occurs in the form of derivatives such as glycosides, amides, and esters. The most numerous groups of caffeic acid derivatives are esters with quinic acid (chlorogenic acid), α-hydroxydihydrocaffeic acid (rosmarinic acid), and tartaric acid (caffeoyltartaric acid), as well as the phenethyl ester of caffeic acid. Caffeic acid monocompound is found in many foods, such as chokeberries, blueberries, apples, plums, spices (thyme, oregano, mint, cinnamon), as well as coffee and red wine. Olives (*O. europaea* L. Oleaceae*, O. europaea fructus*) are a good source of caffeic acid: black (2.10 mg/100 g) and green (1.33 mg/100 g) (Cizmarova et al., 2020). Moreover, it was shown that caffeic acid, both the monocompound and the one released from the esters, have high bioavailability, what offers significant therapeutic potential, especially since caffeic acid is characterized by pleiotropic effects. Studies have confirmed the anti-inflammatory and antioxidant properties of caffeic acid. In human colonic myofibroblasts, caffeic acid downregulates COX-2 expression and reduces PGE2 production (Zielińska et al., 2021). It downregulates inflammatory mediators in endothelial cells (IL-1β, NLRP3, ICAM-1, VCAM-1) (Cao et al., 2019). In ApoE^−/−^ mice, caffeic acid upregulates cholesterol efflux transporters ABCA1 and ABCG1, and promotes HDL-mediated cholesterol removal from macrophages. Moreover, it reduced aortic plaque area by approx. 50% (Sun et al., 2023). Caffeic acid exhibits also metal-ion chelating capacity and radical scavenging ability, contributing to reduced oxidative stress in vascular tissues (Zielińska et al., 2021). This combined antioxidant, anti-inflammatory and lipid-regulatory actions make it a promising dietary phenolic for cardiovascular protection.

#### 3.6.2 Chlorogenic acid

Chlorogenic acid (Table 2) is one of the most common polyphenols found in human food. It can be found in vegetables and fruits such as tomatoes, sweet potatoes, apples, peaches, prunes, oilseeds, green tea, and green coffee. Chlorogenic acid is found particularly in fresh olives (*O. europaea* L., Oleaceae, *O. europaea fructus*) and can enter the oil during the pressing process (Geana et al., 2023). Moreover, fruits and flowers of sweet olives (*O. fragrans* Lour., Oleaceae, *O. fragrans fructus et flos*) are a good source of chlorogenic acid (Fu C. C. et al., 2022). Studies on chlorogenic acid have demonstrated its potent anti-inflammatory and antioxidant properties (Gu et al., 2023; Sato et al., 2011). It has been suggested that, like other polyphenols, chlorogenic acid prevents LDL oxidation, which may inhibit lipid accumulation in macrophages and limit early atherosclerotic plaque growth. Its hypolipidemic effect has been demonstrated (Ong et al., 2013). Moreover, in an ApoE^−/−^ mouse model, chlorogenic acid promoted cholesterol efflux from macrophages by increasing the expression of ATP-binding cassette transporters ABCA1/ABCG1 (Wu et al., 2014).

## 4 Pre-clinical evidence

Despite significant progress in primary prevention and therapeutic strategies, the incidence of atherosclerosis continues to rise, underscoring the urgent need for new therapeutic interventions. A growing number of studies are focusing on natural substances with potential inhibitory properties for the development of heart and vascular diseases. Metabolites found in plants of the Oleaceae family are also relevant in this context (Table 3). Studies in cellular and animal models allow for the assessment of the bioactivity of individual components, especially since their effectiveness may be comparable to or even superior to synthetic drugs.

**TABLE 3:** Mechanisms of the anti-atherosclerotic action of bioactive metabolites.

Bioactive metabolite	Anti-atherosclerotic activity				
	Antioxidant and redox-modulating actions	Anti-inflammatory signaling	Endothelial protection and nitric oxide bioavailabiliy	Lipoprotein oxidation and cholesterol accumulation attenuation	Plaque stability/regression, anti-calcification
Acteoside	↑ Nrf2 activation, ↑ SOD, ↑ CAT, ↑ GPx, ↑ HO-1, ↑ NQO1, ↓ NADPH-oxidase, ↓ ROS activity Yang et al. (2023) Xiao et al. (2022) Wang et al. (2021)	↓ NF-κB pathway, ↓ NLRP3 inflammasom activation, ↓ COX-2/PGE_2_, ↓ iNOS, ↓ IL-1β, ↓ IL-6, ↓ TNF-α secretion, ↓ M1/↑ M2 macrophage polarization Muhtar et al. (2025) Xiao et al. (2022)	↓ ICAM-1, ↓ VCAM-1 activity Chen et al. (2009)	↓ LDL oxidation ↑ cholesterol efflux by ABCA1 transporter Jia et al. (2023) Xiao et al. (2022)	↑ plaque stability/regression Fan and Zhang (2021)
Caffeic acid	↑ Nrf2 activation, ↑ SOD, ↑ CAT, ↑ GPx, ↑ HO-1, ↑ NQO1, ↓ NADPH-oxidase, ↓ ROS activity Wan et al. (2021) Zielińska et al. (2021) Mu et al. (2018)	↓ NF-κB pathway, ↓ NLRP3 inflammasom activation, ↓ COX-2/PGE_2_, ↓ iNOS, ↓ IL-1β, ↓ IL-6, ↓ TNF-α Secretion ↓ M1 macrophage polarization Zhang et al. (2025a) Yu et al. (2024) Kim et al. (2024) Wan et al. (2021) Zielińska et al. (2021) Cao et al. (2019) Khan et al. (2012)	↑ SIRT1, ↓ ICAM-1, ↓ VCAM-1 activity Fleming et al. (2025) Cao et al. (2019)	↓ LDL oxidation, ↑ cholesterol efflux by ABCA1/ABCG1 transporters Sun et al. (2023) Khan et al. (2012)	↑ plaque regression ↓ calcification Chen et al., 2025 Sun et al. (2023)
Chlorogenic acid	↑ Nrf2 activation, ↑ SOD, ↑ CAT, ↑ GPx, ↑ HO-1, ↑ NQO1, ↓ NADPH-oxidase, ↓ ROS activity Dai et al. (2024) Gu et al. (2023) Suzuki et al. (2006)	↓ NF-κB pathway, ↓ NLRP3 inflammasom activation, ↓ COX-2/PGE_2_, ↓ iNOS, ↓ IL-1β, ↓ IL-6, ↓ TNF-α Secretion ↓ M1/↑ M2 macrophage polarization Xue et al. (2024) Gu et al. (2023) Li et al. (2022)	↑ eNOS activation, ↑ SIRT1, ↓ ICAM-1, ↓ VCAM-1 activity Hada et al. (2020) Yuan et al. (2017)	↓ LDL oxidation, ↓ CD36, ↓ intracellular cholesterol accumulation, ↑ cholesterol efflux by ABCA1/ABCG1 transporters Gu et al. (2023) Wu et al. (2014)	↑ plaque regression ↓ calcification Wu et al. (2014) Chen et al. (2023a)
Erythrodiol	↑ GPx Penas-Fuentes et al. (2021)	↓ IL-1β, ↓ IL-6, ↓ TNF-α Secretion Marquez-Martin et al. (2006)	—	↓ LDL oxidation ↑ cholesterol efflux by ABCA1 transporter Wang et al. (2017) Allouche et al. (2010)	↑ plaque regression Cao et al. (2025)
Forsythoside A	↑ Nrf2 activation, ↑ SOD, ↑ CAT, ↑ GPx, ↑ HO-1, ↑ NQO1, ↓ ROS activity Li et al. (2025) Fu et al. (2023)	↓ NF-κB pathway, ↓ NLRP3 inflammasom activation, ↓ COX-2/PGE_2_, ↓ iNOS, ↓ IL-1β, ↓ IL-6, ↓ TNF-α secretion Song et al. (2018) He et al. (2024) Cheng et al. (2014)	↓ ICAM-1, ↓ VCAM-1 activity Sung et al. (2016)	—	—
Forsythoside B	↑ Nrf2 activation, ↑ SOD, ↑ CAT, ↑ GPx, ↑ HO-1, ↑ NQO1, ↓ ROS activity Chen et al. (2023b) Zheng et al. (2021)	↓ NF-κB pathway, ↓ NLRP3 inflammasom activation, ↓ COX-2, ↓ iNOS, ↓ IL-1β, ↓ IL-6, ↓ TNF-α secretion Wang et al. (2025) Xia et al. (2022)	—	↓ LDL oxidation Martin-Nizard et al. (2003)	—
Hydroxytyrosol	↑ Nrf2 activation, ↑ SOD, ↑ CAT, ↑ GPx, ↑ HO-1, ↑ NQO1 ↓ NADPH-oxidase, ↓ ROS activity Wen et al. (2024) Gabbia et al. (2023)	↓ NF-κB pathway, ↓ NLRP3 inflammasom activation, ↓ COX-2/PGE_2_, ↓ iNOS, ↓ IL-1β, ↓ IL-6, ↓ TNF-α Secretion, ↓ M1/↑ M2 macrophage polarization Batarfi et al. (2024) Zodio et al. (2024) Miao et al. (2023) Yonezawa et al. (2018)	↑ eNOS activation, ↑ SIRT1, ↓ ICAM-1, ↓ VCAM-1 activity Vijakumaran et al. (2023) Franceschelli et al. (2023)	↓ LDL oxidation ↑ cholesterol efflux by ABCA1 transporter Del Saz-Lara et al. (2025) Franceschelli et al. (2023)	↑ plaque stability/regression, ↓ calcification Christodoulou et al. (2024) Zoubdane et al. (2024) Zodio et al. (2024)
Ligstroside	↑ Nrf2 activation, ↑ GPx ↑ HO-1, ↓ NADPH-oxidase, ↓ ROS activity Castejon et al. (2022) Grewal et al. (2020)	↓ NF-κB pathway, ↓ NLRP3 inflammasom activation, ↓ COX-2, ↓ iNOS ↓ IL-1β, ↓ IL-6, ↓ TNF-α secretion Kołtun-Jesion et al. (2023) Castejon et al. (2022)	↑ SIRT1 activity Grewal et al. (2020)	↓ LDL oxidation Leenen et al. (2002)	—
Oleacein	↑ Nrf2 activation, ↑ HO-1, ↓ NADPH-oxidase, ↓ ROS activity Filipek and Gierlikowska et al. (2021) Carpi et al. (2019)	↓ NF-κB pathway, ↓ NLRP3 inflammasom activation, ↓ COX-2/PGE_2_, ↓ iNOS, ↓ IL-1β, ↓ IL-6, ↓ TNF-α secretion, ↓ M1/↑ M2 macrophage polarization Rosillo et al. (2024) Gutierrez-Miranda et al. (2020) Filipek et al. (2015)	↓ ICAM-1, ↓ VCAM-1 activity Silvestrini et al. (2023)	↓ LDL oxidation, ↓ CD36, ↓ intracellular cholesterol accumulation, ↑ cholesterol efflux by ABCA1/ABCG1 transporters Filipek and Gierlikowska et al. (2021)	↑ plaque stability Filipek et al. (2017)
Oleanolic acid	↑ Nrf2 activation, ↑ SOD, ↑ CAT, ↑ GPx, ↑ HO-1, ↑ NQO1, ↓ NADPH-oxidase, ↓ ROS activity Wang J. et al. (2024) Jiang et al. (2015) Reisman et al. (2009)	↓ NF-κB pathway, ↓ NLRP3 inflammasom activation, ↓ COX-2/PGE_2_, ↓ iNOS, ↓ IL-1β, ↓ IL-6, ↓ TNF-α Secretion ↓ M1/↑ M2 macrophage polarization Li et al. (2021) Dong et al. (2020) Peng et al. (2019) Hwang et al. (2014)	↑ eNOS activation, ↑ SIRT1 ↓ ICAM-1, ↓ VCAM-1 activity Stelling-Ferez et al. (2023) Peng et al. (2019) Zhang et al. (2018)	↓ LDL oxidation, ↑ cholesterol efflux by ABCA1/ABCG1 transporters Zhang et al. (2016) Jiang et al. (2015)	↑ plaque regression, ↓ calcification Martin et al. (2014) Buus et al. (2011)
Oleoacteoside	↓ ROS activity Dudek et al. (2017)	↓ IL-1β, ↓ IL-6, TNF-α secretion Kołtun-Jasion et al. (2023)	—	—	—
Oleocanthal	↑ Nrf2 activation, ↑ SOD, ↑ GPx, ↑ HO-1, ↓ NADPH-oxidase, ↓ ROS activity Katsa et al. (2024) Montoya et al. (2019)	↓ NF-κB pathway, ↓ NLRP3 inflammasom activation, ↓ COX-2/PGE_2_, ↓ iNOS, ↓ IL-1β, ↓ IL-6, ↓ TNF-α secretion Abdallah et al. (2023) Montoya et al. (2021) Carpi et al. (2019)	↑ eNOS activation Montoya et al. (2023)	↓ LDL oxidation ↑ cholesterol efflux by ABCA1 transporter Katsa et al. (2024) Qosa et al. (2015)	↑ plaque stability/regression Christodoulou et al. (2024)
Oleuropein	↑ Nrf2 activation, ↑ SOD, ↑ CAT, ↑ GPx, ↑ HO-1, ↑ NQO1, ↓ NADPH-oxidase, ↓ ROS activity Hu et al. (2025) Delle Monache et al. (2021) Sun et al. (2017b) Romero et al. (2016)	↓ NF-κB pathway, ↓ NLRP3 inflammasom activation, ↓ COX-2/PGE_2_, ↓ iNOS, ↓ IL-1β, ↓ IL-6, ↓ TNF-α secretion, ↓ M1/↑ M2 macrophage polarization Silvestrini et al. (2023) Mirsanei et al. (2023) Castejon et al. (2019)	↑ eNOS activation, ↑ SIRT1, ↓ ICAM-1, ↓ VCAM-1 activity Hu et al. (2025) Silvestrini et al. (2023) Romero et al. (2016)	↓ LDL oxidation Bertin et al. (2016)	↓ calcification Banaei et al. (2025)
Phillygenin	↑ Nrf2 activation, ↑ SOD, ↑ CAT, ↑ GPx, ↑ HO-1, ↑ NQO1, ↓ ROS activity Wei et al. (2025) Guo et al. (2022a)	↓ NF-κB pathway, ↓ NLRP3 inflammasom activation, ↓ COX-2/PGE_2_, ↓ iNOS, ↓ IL-1β, ↓ IL-6, ↓ TNF-α Secretion, ↓ M1 macrophage polarization Wang Y. Q. et al. (2024) Ma et al. (2023) Guo et al. (2022b)	—	↓ LDL oxidation ↑ cholesterol efflux by ABCA1 transporter Filipek et al. (2025)	↑ plaque stability Filipek et al. (2025)
Syringin	↑ Nrf2 activation, ↑ SOD, ↑ CAT, ↑ GPx, ↑ HO-1 ↑ NQO1, ↓ ROS activity Zhao et al. (2023) Wang et al. (2020)	↓ NF-κB pathway, ↓ NLRP3 inflammasom activation, ↓ COX-2/PGE_2_, ↓ iNOS, ↓ IL-1β, ↓ IL-6, ↓ TNF-α Secretion ↓ M1 macrophage polarization Cong et al. (2025) Arshad et al. (2024) Zhao et al. (2023) Fu R. H. et al. (2022)	↑ SIRT1 ↓ ICAM-1, ↓ VCAM-1 activity Zhao et al. (2025), (2023) Nan et al. (2024)	↓ LDL oxidation; ↓ CD36, ↓ intracellular cholesterol accumulation Filipek et al., 2025	↑ plaque stability Filipek et al. (2025) Nan et al. (2024)
Ursolic acid	↑ Nrf2 activation, ↑ SOD, ↑ CAT, ↑ GPx, ↑ HO-1, ↑ NQO1, ↓ NADPH-oxidase, ↓ ROS activity Yu et al. (2017) Ma et al. (2015)	↓ NF-κB pathway, ↓ NLRP3 inflammasom activation, ↓ COX-2/PGE_2_, ↓ iNOS, ↓ IL-1β, ↓ IL-6, ↓ TNF-α Secretion ↓ M1/↑ M2 macrophage polarization Lei et al. (2023) Wang et al. (2023) Ma et al. (2015)	↑ eNOS activation, ↑ SIRT1, ↓ ICAM-1, ↓ VCAM-1 activity Pordanjani et al. (2022) Lin et al. (2016)	↓ LDL oxidation Jiang et al. (2016)	↑ plaque regression Li et al. (2018)

### 4.1 *In vitro* models: endothelial cells, macrophages, vascular smooth muscle cells

Although elevated lipid levels were once considered a major driving factor, it is now widely accepted that atherosclerosis is fundamentally an inflammatory condition in which all stages of plaque development are closely linked to inflammatory responses. The inflammatory component, characterized by the involvement of various immune cells and the release of cytokines, contributes significantly to the complexity and progression of the disease (Libby et al., 2002). Therefore, strategies targeting the inflammatory cascade may represent one promising avenue for therapeutic intervention. All of the above-described active metabolites of plants from the Oleaceae family exhibit potent anti-inflammatory properties by inhibiting the production of inflammatory cytokines such as IL-6, IL-1β, and TNF-α. Moreover, these same metabolites possess potent antioxidant properties by reducing ROS production *via* the Nrf2 pathway (Table 3).

Mitigating inflammation and oxidative stress modulates lipid metabolism, which is crucial in both early and late atherosclerotic lesions. In in vitro, as well as *in vivo* models, Oleaceae metabolites demonstrated a reduction in LDL oxidation, which is directly related to endothelial damage and the formation of foam cells from lipid-laden macrophages (Mannarino and Pirro, 2008; Zong et al., 2022). Cholesterol efflux from foam cells occurs *via* an active pathway involving ATP-binding cassette transporters (ABCA1, ABCG1) and contributes to the regression of atherosclerosis in its early stages. In in vitro studies, chlorogenic acid (Gu et al., 2023), erythrodiol (Wang et al., 2017), hydroxytyrosol (Franceschelli et al., 2023), oleacein (Filipek and Gierlikowska, 2021), oleanolic acid (Zhang et al., 2016), oleocanthal (Qosa et al., 2015) and phillygenin (Filipek et al., 2025) enhance reverse cholesterol transport by activating transporters ABCA1 or/and ABCG1. In turn, chlorogenic acid (Gu et al., 2023), oleacein (Filipek et al., 2020) and syringin (Filipek et al., 2025) have been shown to inhibit the expression of the CD36 receptor, which promotes cholesterol uptake by macrophages, and under pathophysiological conditions leads to the formation of foam cells and atherosclerotic plaque.

Given that the integrity of the fibrous cap is crucial for preventing atherosclerotic plaque rupture and subsequent thrombotic complications, natural compounds that enhance collagen synthesis or inhibit its degradation are of particular interest. Inflammatory mediators are known to reduce the synthesis of interstitial collagen, a product of smooth muscle cells in the arterial wall that strengthens the fibrous cap of atherosclerotic plaque. In this sense, the described metabolites from the Oleaceae family may stabilize collagen production by reducing inflammation. However, stable interstitial collagen depends not only on the rate of its synthesis but also on its catabolism. Interstitial collagenases, members of the matrix metalloproteinase (MMP) family, can initiate proteolytic cleavage of the stable collagen triple helix. This cleavage by MMPs initiates the collagen catabolic cascade. Interstitial collagenases, belonging to the matrix metalloproteinase (MMP) family, can initiate proteolytic cleavage of the stable collagen triple helix. This cleavage by MMPs initiates the collagen catabolic cascade. Under physiological conditions, arteries do not produce MMPs. However, proinflammatory M1 macrophages present in atherosclerotic plaque promote increased expression of extracellular matrix proteins (Visse and Nagase, 2003). Filipek et al. (2017) demonstrated that oleacein inhibits the secretion of MMP-9 gelatinase and the MMP-9/neutrophil gelatinase-associated lipocalin complex (MMP-9/NGAL) in atherosclerotic plaque obtained from human endarterectomy. This oleacein activity promotes plaque stabilization.

An important mediator of the inflammatory response that induces the expression and secretion of MMP-9 in macrophages is prostaglandin E2, which is produced in the arachidonic acid pathway *via* the cyclooxygenase enzymes COX-1 and COX-2. COX-1 is known to be present in most tissues and is involved in maintaining homeostasis under physiological conditions. On the other hand, COX-2 is activated during the inflammatory reaction, when an immune response develops. Therefore, it appears at sites of inflammation (e.g., atherosclerotic plaque) and is induced by cytokines such as IL-1 β, IL-6, and TNF-α (Pavlovic et al., 2006). Additionally, arachidonic acid metabolism leads to the formation of thromboxane (A2, B2), which increase platelet aggregation and may enhance the formation of arterial thrombi. It is also important to remember that the primary regulator of inflammation in all stages of atherosclerosis is nuclear factor kappa B (NF-κB), which is responsible for regulating proinflammatory markers such as chemokines, cytokines (IL-1β, IL-6, TNF-α), adhesion molecules (ICAM-1, VCAM-1), the COX-2 enzyme, inducible nitric oxide synthase (iNOS), as well as apoptosis and cellular proliferation (De Winther et al., 2005).

As previously mentioned, all Oleaceae metabolites have been shown to reduce the secretion of pro-inflammatory cytokines (Table 3). *In vitro* studies have shown that acteoside (Muhtar et al., 2025), caffeic acid (Kim et al., 2024), chlorogenic acid (Xue et al., 2024), forsythoside A (Song and Lei, 2025), forsythoside B (Xia et al., 2022), hydroxytyrosol (Yonezawa et al., 2018), oleacein (Gutierrez-Miranda et al., 2020), oleanolic acid (Dong et al., 2020), oleocanthal (Carpi et al., 2019), oleuropein (Mirsanei et al., 2023), phillygenin (Ma et al., 2023), syringin (Arshad et al., 2024) and ursolic acid (Lei et al., 2023) inhibited NF-κB expression, which presumably reduced inflammation induced by various proinflammatory markers (IL-1β, IL-6, TNF-α, COX-2, iNOS). Furthermore, the same metabolites also reduced COX-2 secretion in the arachidonic acid/prostaglandin E2 pathway.

Beyond these direct mechanisms, the role of macrophages, specifically their polarization into proatherogenic M1 and antiatherogenic M2 phenotypes, represents another key therapeutic target for natural metabolites from plants of the Oleaceae family. M1 macrophages are known to secrete proinflammatory cytokines that exacerbate atherosclerotic plaque progression. Furthermore, chronic inflammation is closely linked to endothelial dysfunction and the activation of transcription factors, which together perpetuate a positive feedback loop, driving the development and progression of atherosclerosis (Zhang and Dhalla, 2024). Acteoside, caffeic acid, chlorogenic acid, forsythoside A, hydroxytyrosol, oleacein, oleanolic acid, oleuropein, syringin and ursolic acid inhibit the expression of intercellular adhesion molecules (ICAM-1, VCAM-1) found on the surface of endothelial and immune cells (macrophages, lymphocytes) (Table 3). This prevents the migration and adhesion of proinflammatory cells, which consequently damage the endothelium and promote the progression of early atherosclerotic lesions. On the other hand, in a cellular model, Filipek et al. (2015) demonstrated that oleacein can switch macrophages from M1 to M2, which was associated with increased expression of the IL-10 receptor and CD 163. Furthermore, in advanced atherosclerotic plaques, oleacein inhibited macrophage apoptosis and increased the secretion of high mobility group B1 proteins (HMGB1), which are markers of ischemia and cell damage associated with the risk of cardiovascular death in patients with acute coronary syndrome (Filipek et al., 2017; Filipek and Gierlikowska, 2021). In the RAW 264.7 cell model, Mirsanei et al. (2023) showed that oleuropein decreased the expression of M1-related cytokines (IL-12, IFN-γ, and TNF-α) and gene expression (iNOS, TNF-α), while increasing the expression of M2-related anti-inflammatory gene and cytokine production (IL-10 and TGF-β). It is important to remember that macrophage phenotypes, particularly M1 (proinflammatory) and M2 (anti-inflammatory), are key determinants of atherosclerotic plaque progression and stability (Sitaula et al., 2015). Dysregulation of macrophage function, and in particular an imbalance between these phenotypes, can lead to impaired tissue repair and persistent inflammation, further contributing to the development of atherosclerotic plaques.

The vascular endothelium plays a key role in atherosclerosis, as its dysfunction is the basis for the development of this disease. Hydroxytyrosol at a concentration of 50 μM was shown to increase NO production by activating eNOS in endothelial cells derived from porcine arteries. In contrast, a two-fold increase in hydroxycortisol concentration inhibited the production of oxidative stress markers, malondialdehyde (MDA) and ROS, thereby increasing superoxide dismutase (SOD) production in TNF-induced HUVEC cells. Furthermore, hydroxytyrosol is a mitochondrial ROS scavenger in PMA-induced HUVEC cells. This compound may also reduce mitochondrial superoxide production by increasing SOD activity (Vijakumaran et al., 2023).

### 4.2 *In vivo* models: ApoE^−/−^ mice, LDL-R^−/−^ mice, diet-induced atherosclerosis

The ApoE *in vivo* model is a genetic assay or model organism used to study the role of the ApoE gene, which is crucial for lipid metabolism and a significant risk factor for atherosclerosis. ApoE −/− mice have impaired lipoprotein metabolism, leading to the accumulation of lipid deposits and the development of atherosclerosis. Other animal models, particularly LDL-R−/− mice, are known to be crucial for assessing the *in vivo* efficacy of natural compounds. Studies often involve long-term dietary interventions to induce significant atherosclerotic plaque formation.

It seems that the best-studied *in vivo* metabolites of Oleaceae so far are the most well-known ones, such as: hydroxytyrosol, oleuropein, oleacein, oleaocanthal and oleanolic acid. Zhang et al. (2020) showed that hydroxytyrosol at a dose of 10 mg/kg body weight reduced the levels of total cholesterol, LDL-C and triglycerides in both the serum and liver of ApoE^−/−^ mice. Simultaneously, an increase in HDL-C concentration was observed, contributing to the reversal of hepatic steatosis. The same team of researchers also elucidated the mechanism of hydroxytyrosol’s antiatherosclerotic action. They discovered that hydroxytyrosol can regulate AMPK/SREBP2 signaling and enhance the expression of SRB1, ABCA1, and apoAI receptors, responsible for lipid efflux from foam cells. Furthermore, they confirmed that hydroxytyrosol modulates cholesterol metabolism by reducing p38 phosphorylation and subsequently activating AMPK and inactivating NF-κB. This, in turn, led to the blockade of SREBP2/PCSK9 and the increased expression of LDLR, apoAI, and ABCA1, ultimately leading to a reduction in LDL-C and an increase in HDL-C concentrations in the circulation. On the other hand, in ApoE^−/−^ mice fed a Western-type diet for 20 weeks, hydroxytyrosol reduced the development of atherosclerotic lesions in the aortic arch and also inhibited the activity of mediators of oxidative stress (NADPH oxidase subunits: NOX2 and p22phox) and inflammation (IL-1β and MCP-1) (Hara et al., 2023). Furthermore, hydroxytyrosol was shown to reduce the secretion of proinflammatory cytokines (CRP, TNF-α, IL-1β, and IL-6) while simultaneously increasing IL-10 (Zhang et al., 2020). In another study, hydroxytyrosol in ApoE^−/−^ mice fed a high-fat diet (HFD) for 12 weeks was shown to have a protective effect on the vascular endothelium by inhibiting pyroptosis in the aortic intima *via* the class IV histone deacetylase 11 (HDAC11)-related signaling pathway (Yao et al., 2021).

Christodoulou et al. (2024) evaluated the cardioprotective properties of hydroxytyrosol, oleuropein, oleocanthal, and oleanolic acid as single components and in combination therapy. They found that oleocanthal, oleanolic acid, and oleuropein, but not hydroxytyrosol, significantly reduced infarct size *in vivo* compared with the control group. Oleuropein exhibited antihyperglycemic properties, and oleanolic acid effectively alleviated hypercholesterolemia. The combination regimens oleuropein + hydroxytyrosol + oleanolic acid, oleuropein + hydroxytyrosol + oleocanthal, and oleuropein + hydroxytyrosol + oleanolic acid + oleocanthal were cardioprotective. However, only oleuropein + hydroxytyrosol + oleocanthal demonstrated effective relief of hyperglycemia, suppression of apoptosis, enhancement of antioxidant activity, and increased expression of antioxidant enzymes. Moreover, this combination regimen reduced the extent of atherosclerotic plaque in ApoE^−/−^ mice.

Another antiatherosclerotic mechanism of oleuropein has also been suggested. In apoE knockout mice, oleuropein bound to peroxisome proliferator-activated receptors (PPARs) in distinct conformations, depending on the way PPAR agonists bind to their active sites (Huang et al., 2010).

Caffeic acid and its derivative, chlorogenic acid, have been quite well studied in the ApoE^−/−^ mouse model. Two-month treatment with chlorogenic acid at a dose of 400 mg/kg body weight also significantly reduced the burden of atherosclerotic lesions in the aortas. These effects were associated with increased HDL-C (caffeic acid) concentrations as well as decreased serum levels of total cholesterol, triglycerides, and LDL-C. Both metabolites promoted increased expression of ABCA1 and ABCG1 transporter proteins, as well as PPAR-γ and LXRα transcriptional activity. In addition to the increased cholesterol outflow from foam cells, a decreased secretion of proinflammatory cytokines (TNF-α, IL-6 and MCP-1) was also demonstrated (Wu et al., 2014; Sun et al., 2023). Similarly, in ApoE^−/−^ mice fed an atherogenic diet supplemented with ursolic acid (100 mg/kg/day), atherosclerotic plaque formation was significantly reduced, and the necrotic core areas of the atherosclerotic plaque were reduced. In the case of ursolic acid, this effect was achieved by inhibiting LOX-1 *via* the ROS/NF-κB signaling pathway. Additionally, in this study, the bioactivity of ursolic acid was comparable to that of the synthetic drug - simvastatin (Li et al., 2018). Furthermore, Ma et al. (2022) demonstrated that ursolic acid inhibited the catalytic activity of hydroxy-3-methylglutaryl-coenzyme A synthetase 1 (HMGCS1) by irreversibly binding to the thiol Cys-129, which consequently reduced the production of precursors for cholesterol biosynthesis *in vivo*. Moreover, this metabolite reduced the atherosclerotic area in the entire aorta of mice with diet-induced hypercholesterolemia.

Other Oleaceae metabolites, i.e.,: acteoside, erythrodiol, forsythoside A, forsythoside B, ligstroside, syringin and phillygenin, have not yet been tested on animal models, although promising *in vitro* studies suggest their use in the treatment of cardiovascular diseases.

### 4.3 Bioavailability of metabolites from the Oleaceae family

The Absorption of Oleaceae metabolites is a complex process and depends on various factors, such as molecular weight, polarity, degree of polymerization (Carbonell-Capella et al., 2014), solubility, lipophilicity, and phenol-binding capacity. It is known that the absorption and metabolism of compounds determine their health potential. Small-molecule compounds such as hydroxytyrosol and caffeic acid are readily absorbed from the small intestine into the bloodstream and distributed to tissues. The main pathways of hydroxytyrosol metabolism are conjugation with glucuronic acid and sulfuric acid (glucuronidation and sulfation). The resulting metabolites, such as hydroxytyrosol-3-O sulfate (HT-3-S), homovanillic acid (HVA), and dihydroxyphenylacetic acid (DOPAC), not free hydroxytyrosol, are responsible for many of the beneficial effects observed in studies. Hydroxytyrosol and its metabolites are rapidly excreted by the kidneys, meaning that hydroxytyrosol does not accumulate in the body in significant amounts. The bioavailability and metabolic fate of hydroxytyrosol depend on the food matrix in which it is consumed, and extra virgin olive oil has been found to be a particularly effective carrier (Bender et al., 2023). Caffeic acid is also well absorbed in the small intestine (95%). The bioavailability of caffeic acid may be higher when consumed in its free form than in its bound form (e.g., as chlorogenic acid). Its absorption may be dependent on the gut microflora, specifically the presence of ferulic acid esterases. After absorption, it is metabolized in the body by various pathways, including methylation, sulfation, or glucuronidation. Caffeic acid metabolites (ferulic and benzoic acid derivatives) can be excreted in urine and bile (Cizmarova et al., 2020).

The bioavailability of higher molecular weight compounds (>200 kDa) varies. Song and Lei (2018) demonstrated *in vivo* (mice) that phillygenin was effectively absorbed after oral administration, with an oral bioavailability of 56.4%. Additionally, metabolites: hydroxylated and dimethylated phillygenin were detected in mouse urine. The bioavailability of oleacein may be equally high. Oleacein has been found to be stable at gastric acid pH, with 67% remaining unchanged after 4 h of incubation (Shimamoto et al., 2023). Oleacein is very similar to oleocanthal, as the latter differentiates with one fewer -OH group at the C-3 carbon of the phenylalcohol moiety. Both molecules are quite sensitive and unstable, which translates into analytical difficulties. This explains the limited and often contradictory pharmacokinetic studies. Few studies have confirmed that the intestinal permeability of oleacein is comparable to that of the highly permeable compound naproxen (Lozano-Castellón et al., 2020). During absorption, oleacein undergoes significant metabolism, primarily through phase I reactions (hydrolysis and oxidation), and metabolites occur at higher concentrations in plasma than in the intestinal lumen. This suggests a significant “first-pass effect” in the intestine. The main circulating metabolites are those formed by hydrolysis (hydroxytyrosol) and hydroxylation (oleacein + OH). Oleacein is also metabolized *via* glucuronidation, a phase II reaction in which glucuronic acid is added to the molecule. Glucuronidated metabolites can be released from glucuronides by lysosomal beta-glucuronidase at sites of inflammation (López-Yerena et al., 2021). Even less data exists for oleocanthal. Oleocanthal is presumed to be poorly absorbed in the intestine and undergoes extensive metabolism, primarily through phase I reactions. These reactions include hydration, hydrogenation, and hydroxylation. Phase II reactions, such as glucuronidation, also occur, particularly in plasma (Lozano-Castellón et al., 2020). Like oleocanthal, oleuropein is also poorly absorbed from the small intestine and is characterized by low bioavailability (Edgecombe et al., 2000). The mechanism of this process is still not fully understood, but it may involve paracellular movement (movement through tight junctions) or transcellular transport. It is believed that oleuropein undergoes extensive metabolism to hydroxytyrosol and tyrosol, as well as other products such as elenolic acid and glucose. Kendall et al. (2009) demonstrated that the glucose transporter is involved in the oleuropein absorption process. Another theory assumes that oleuropein is a stable molecule and reaches the large intestine unchanged, where biotransformation occurs by intestinal microflora. Hydroxytyrosol is formed and then released into the bloodstream. Deglycosylation of oleuropein also occurs in the large intestine, leading to the formation of aglycone forms through the action of β-glycosidase (Žugcic et al., 2019).

The bioavailability of the remaining Oleaceae metabolites administered *per os, i.e.*, intact chlorogenic acid, ursolic acid, oleanolic acid and syrigin, is also low, ranging from 5% to 10%. *In vivo* studies (Wistar rats) have confirmed that chlorogenic acid, like oleuropein, is metabolized by the gut microbiome to phenolic acids such as m-coumaric acid, 3-hydroxyphenylpropionic acid and hippuric acid which are then absorbed and potentially exert biological effects (Gonthier et al., 2003). Similarly, syringin is rapidly eliminated. Yang et al. (2022) found that rat gut flora metabolized more than 80% of syringin within 12 h, and erucic alcohol was identified as the major metabolite. Syringin itself was not detected in plasma or bile, suggesting low bioavailability. On the other hand, other forms of administration, such as injection, significantly increase the bioavailability of this compound, as determined by Qian et al. (2024) in rat plasma. The AUC0−∞ values of syringin were (429.5 ± 25.6) and (721.0 ± 81.8) µg/h/L, respectively, and the plasma clearance (CL) of syringin was (3.3 ± 0.2) and (2.0 ± 0.2) L/h/kg, respectively, with both values showing significant differences.

Moreover, ursolic and oleanolic acids are characterized by low oral absorption and bioavailability due to poor water solubility, which may limit their therapeutic use. Increased absorption of these metabolites can be achieved by co-administration with edible oils or through innovative technologies such as lipid nanoparticles (Staicu et al., 2024). Data on other Oleaceae metabolites are quite limited. It is known that acteoside can be hydrolyzed to degradation products, particularly caffeic acid and hydroxytyrosol (Xiao et al., 2022). However, oleoacteoside, forsythioside A and forsythioside B still require research on their bioavailability and metabolism.

The low bioavailability of Oleaceae metabolites is the main limitation in their use as natural medicines. One technique for increasing the bioavailability of metabolites is their structural modification. Several modification methods are known, including chemical, enzymatic, and microbiological. Due to its relatively high selectivity while maintaining mild conditions, the enzymatic method is becoming increasingly popular. Metabolites from the Oleaceae family can be a valuable resource for the development of new drugs. Furthermore, cataloguing such modified and structurally optimized molecules can create a vast collection of natural products and increase the potential for developing new drugs. This field has advanced significantly with the advent of bioinformatics technologies, including artificial intelligence (AI) and advanced computing. Few studies report modification of ursolic acid and oleanolic acid to improve their bioavailability. Structural modifications of ursolic acid and oleanolic acid involve primarily chemical and microbiological methods, altering the C-3 hydroxyl group, the C-28 carboxylic acid, and the C-12-C-13 double bond in the case of ursolic acid. This modification results in the formation of esters and amides, and additionally, oleanolic acid oxime at the C3 position. These compounds in the new configuration are characterized not only by improved bioavailability but also by greater water solubility and potency (Mlala et al., 2019; Zhou et al., 2026).

### 4.4 Pharmacodynamic correlations

It is known that the bioavailability and metabolism of Oleaceae metabolites determine their pharmacodynamic effect. It appears that hydroxytyrosol, which is commonly found in various plant raw materials and products, and can also be formed as a result of the transformation of oleuropein, oleacein, and oleocanthal, appears to have the strongest antioxidant activity (Edgecombe et al., 2000; López-Yerena et al., 2021; Lozano-Castellón et al., 2020). By neutralizing ROS, hydroxytyrosol protects cells from oxidative damage and enhances the activity of antioxidant mediators such as superoxide dismutase (SOD) and E2-related nuclear factor 2 (Nrf2). Nrf2 is known to be one of the most potent antioxidant pathways that prevents oxidative damage in vascular endothelial cells. Hydroxytyrosol at a concentration of 50 µM induced repair processes by increasing the expression of Nrf2 and heme oxygenase (HO-1) in the phosphatidylinositol 3-kinase/serine-threonine kinase 1 (PI3K/Akt) and extracellular signal-regulated kinase 1/2 (ERK1/2) signalling pathways (Montoya et al., 2018). Apart from hydroxytyrosol, by enhancing Nrf2/HO-1 activity, oleacein and oleuropein protected endothelial progenitor cells from damage by angiotensin 2 (Parzonko et al., 2013).

The Nrf2/HO-1 pathway is also characteristic of ligstroside bioactivity. Castejon et al. (2022) showed that increased Nrf2/HO-1 secretion resulted in decreased markers of oxidative stress, such as NO production, inducible nitric oxide synthase (iNOS), and NADPH oxidase-1 (NOX-1) protein expression. However, in the case of ligstroside, further studies are needed to clarify the protective effects of this compound.

Another important role of the Nrf2/HO-1 pathway is to protect cholesterol from oxidation (oxLDL), which prevents the deposition of lipid deposits in macrophages and limits the formation of foam cells, which contribute to atherosclerotic plaque. This activity has been confirmed for oleacein (Filipek and Gierlikowska, 2021) and phillygenin (Filipek et al., 2025).

Activation of the Nrf2/HO-1 pathway by another metabolite - syringin - led to the repair of heart muscle cells, which improved heart function and reduced the extent of the infarction (Zhao et al., 2023). Unfortunately, Filipek et al. did not confirm the activation of the Nrf2/HO-1 pathway by syringin.

In recent years, considerable attention has been focused on the Nrf2/Keap1 pathway as a marker associated with the occurrence of cardiovascular disease. Zhang et al. (2025b) demonstrated that caffeic acid and chlorogenic acid bind to Keap1. Both metabolites induce conformational changes in Keap1 by interacting with residues M550 and N532, which subsequently activates Nrf2. Here, too, reducing oxidative stress is associated with the inhibition of cholesterol oxidation, which may limit the development of early atherosclerotic changes.

Other Oleaceae metabolites also demonstrate activation of Nrf2-dependent pathways. Although research focuses on different models, it can be assumed that these compounds may have a protective effect in the early and advanced stages of atherosclerosis.

Sirtuin signaling pathway cascade 1 (SIRT1) plays a protective role in atherosclerosis. Sirtuins (including SIRT1) are a family of enzymes that require NAD+ to function as histone deacetylases. Their protective mechanisms in atherosclerosis involve several pathways. By activating endothelial nitric oxide synthase (eNOS), sirtuins promote NO production, which improves vascular function and reduces plaque development. Sirtuins may block inflammatory processes by inhibiting pathways such as NF-κB, a key driver of the chronic inflammation seen in atherosclerosis. Additionally, SIRT1 improves endothelial function and prevents the expression of endothelial adhesion molecules, which contribute to the formation of atherosclerotic plaques. It plays a key role in protecting vascular smooth muscle cells (VSMCs) from DNA damage and aging (Gorenne et al., 2012). Among the Oleaceae metabolites discussed, the intensification of SIRT1 pathway activity was demonstrated for: caffeic acid (Fleming et al., 2025), chlorogenic acid (Hada et al., 2020), hydroxytyrosol (Vijakumaran et al., 2023), ligstroside (Grewal et al., 2020), oleanolic acid (Stelling-Ferez et al., 2023), oleuropein (Hu et al., 2025), syringin (Zhao et al., 2023) and ursolic acid (Pordanjani et al., 2022).

It is known that nuclear factor kappa B (NF-κB) is an excellent marker of ongoing inflammation. It plays a key role in the development of atherosclerosis by activating inflammatory processes in the blood vessel wall. Its activation increases the expression of proinflammatory genes such as TNF-α, IL-1β, IL-6, and MCP-1, which lead to the retention of inflammatory cells (e.g., macrophages M1) and the formation and development of unstable atherosclerotic plaque. Therefore, inhibition of NF-κB may have protective and antiatherosclerotic effects. Strong anti-inflammatory properties involving the NF-κB pathway have been demonstrated in scientific works for virtually all Oleaceae metabolites, excluding only erythrodiol and oleoacteoside. In addition, oleacein may reduce inflammation by changing the macrophage phenotype from M1 (pro-inflammatory) to M2 (anti-inflammatory), protecting the atherosclerotic plaque from rupture in advanced stages of atherosclerosis (Filipek et al., 2015; Filipek et al., 2017; Filipek and Gierlikowska, 2021). It is known that the switch of macrophages from M1 to M2 depends on the activation of the JAK/STAT 3 pathway. Such biological activity has been demonstrated for oleacein (Filipek et al., 2020).

### 4.5 Limitations and heterogeneity of *in vitro* and *in vivo* studies

Although *in vitro* studies provide information on the mechanisms of action, they have revealed certain limitations that hinder the comparison of individual metabolites. The doses used varied, with the most common range being 10–100 μM. For some metabolites, lower concentrations were used (e.g., erythrodiol, forsythosides, <50 μM). Some investigations assessed only two concentrations, preventing the construction of full dose-response curves. In most cases, approximate minimal effective concentrations could only be inferred from significance thresholds rather than being determined experimentally. Experimental models for studying endpoints related to atherosclerosis were mainly based on THP-1 macrophage-derived foam cells, while LPS-stimulated RAW264.7 macrophages were used to study inflammatory signaling. However, almost all studies relied on single cell lines, limiting generalizability and excluding endothelial-macrophage and multicellular interactions. The exposure time was usually 24 h, which is the accepted standard for testing anti-atherosclerotic activity. However, control conditions were a consistent limitation. While all studies included appropriate negative controls, positive controls were frequently absent. Comparators were only used in a few cases, such as dexamethasone in the syringin study (Arshad et al., 2024) and kaempferol in the syringin, phillygenin, and oleacein studies (Filipek et al., 2025, Filipek and Gierlikowska, 2021).

*In vivo* studies on metabolites derived from the Oleaceae family have reported positive results, such as reduced atherosclerotic plaque burden, improved lipid profiles, and reduced vascular inflammation. However, the pharmacological rigor of these studies varies considerably. For example, some studies employed a single fixed dose without examining dose-response relationships. The animal models used were appropriate for atherosclerosis research, with most studies relying on ApoE −/− mice fed a high-fat diet. While these models collectively reflect key disease processes, their heterogeneity, including differences in diet composition, animal age, and timing of intervention, limits comparability between studies. Control conditions were generally adequate. However, only a few studies, such as the chlorogenic and ursolic acid study, which used atorvastatin or simvastatin as a comparator (Wu et al., 2014; Li et al., 2018), allowed for comparison with established therapies. Regarding exposure durations, interventions typically lasted between 4 and 20 weeks, varying in their preventive or therapeutic intent. The assessed pharmacodynamic endpoints, including atherosclerotic plaque area, lipid profiles, systemic cytokines, endothelial pyroptosis, and hepatic lipid metabolism, were relevant and consistent across studies. However, the lack of pharmacokinetic analyses in nearly all studies limits understanding of exposure-response relationships, especially since some doses exceed anticipated physiological levels.

## 5 Clinical evidence

### 5.1 Randomized controlled trials correlating the polyphenol content of olive oil with cardiovascular disease outcomes

Clinical studies examine the safety and health effects of both mono-nutrients and food ingredients containing these compounds. Many clinical studies focus on extra virgin olive oil (EVOO) due to its highest content of bioactive polyphenols. These studies mainly examine EVOO’s effects on body composition, liver function, and the gut microbiome, as well as measuring inflammatory and oxidative markers. Early results from large, randomized trials, such as PREDIMED, show that a diet rich in extra virgin olive oil can significantly reduce the risk of all-cause and cardiovascular mortality. A 10-year cohort study of a Mediterranean population (>12,000 people) found that moderate daily consumption of extra virgin olive oil (1.5 tablespoons) was associated with a one-third reduction in the risk of all-cause mortality and a half-reduction in the risk of cardiovascular mortality. These effects were not observed with so-called “ordinary” olive oil (Donat-Vargas et al., 2023).

Other multicenter and long-term cohort studies on numerous study groups (AWHS - 2,318 men, SUN project - 18,266 men and women, EPIC-Spain cohort - 39,393 men and women) have shown that the consumption of extra virgin olive oil (2 tablespoons per day) significantly reduces the risk of developing coronary heart disease and stroke. Computed tomography studies have shown a reduction in calcium deposits in atherosclerotic and coronary plaques, which has been linked to the properties of extra virgin olive oil in preventing early-stage plaque development (Donat-Vargas et al., 2022).

Coronary Diet Intervention with Olive Oil and Cardiovascular Prevention (CORDIOPREV) study, conducted over 7 years, assessed the effect of a Mediterranean diet rich in extra virgin olive oil on cardiovascular events such as revascularization, myocardial infarction, peripheral artery disease, stroke, and cardiovascular death in people (approximately 1,000 people) with coronary heart disease. The study results clearly demonstrated a reduction in cardiovascular events. In this regard, the Mediterranean diet proved more effective in secondary prevention than a low-fat diet (Delgado-Lista et al., 2022).

Improvement in vascular endothelial dysfunction was demonstrated in the additional CORDIOPREV study. The study group included patients with type 2 diabetes, prediabetes, and nondiabetic patients. Again, the Mediterranean diet resulted in vasodilation and increased blood flow in both prediabetes and diabetic patients (Daimiel et al., 2020). The reduction of endothelial dysfunction has also been demonstrated in other clinical studies. Valls et al. (2015) conducted randomized controlled trial in patients with hypertension. The researchers observed that just 2 hours after consuming polyphenolic olive oil, the patients experienced increased ischemic reactive hyperemia, and a secondary metabolite (hydroxytyrosol sulfate) appeared in the blood serum. Furthermore, the concentration of ox-LDL, which under pathophysiological conditions damages the cells that form the inner walls of blood vessels, causing their dysfunction, decreased. On a small study group (21 individuals) demonstrated that consuming a single meal containing high-polyphenol olive oil increased the metabolite nitric oxide (NOx) and decreased the levels of lipoperoxides (LPO) and 8-epi prostaglandin-F2α. A positive correlation was also found between NOx and improved endothelial function, whereas negative correlations were observed between ischemic reactive hyperemia (IRH) and LPO and 8-epi prostaglandin-F2α levels (Ruano et al., 2005). In turn, Sanchez-Rodriguez et al. (2018) in a randomized, double-blind, controlled study assessed the effect of extra virgin olive oil supplemented with polyphenols and triterpenoids on metabolic syndrome and vascular endothelial biomarkers. After 3 weeks of consuming different olive oil variants, a significant increase in HDL fraction and a decrease in plasma endothelin-1 were observed in the phenolic-rich olive oil. At the same time, the therapeutic effect was not correlated with triterpenoid content. In the next study, Visioli et al. (2005) demonstrated a vasoprotective effect of extra virgin olive oil in patients with mild dyslipidemia. Furthermore, a decrease in serum thromboxane B2 (TXB2) concentration and an increase in plasma antioxidant activity (8-iso-PGF2alpha) were also observed in the group treated with refined olive oil. However, a significant increase in cardiovascular markers was observed only in the group consuming extra virgin olive oil. On the other hand, Sarapis et al. (2020) compared the effects of high- and low-polyphenol olive oils on blood pressure and arterial stiffness in healthy Australian adults. Fifty participants in this study consumed four tablespoons of the respective olive oil for 3 weeks. It is found that only the high-polyphenol olive oil reduced systolic blood pressure, supporting the effectiveness of dietary intervention in the treatment of cardiovascular disease. Similar results were obtained by Fito et al. (2005) in a Spanish population. A study group (40 men) with stable coronary artery disease took either extra virgin olive oil (high polyphenol content) or filtered olive oil (low polyphenol content) for two sets of 3-week periods, with 2-week breaks in which only filtered olive oil was administered. The result was a reduction in systolic blood pressure, particularly in those with baseline hypertension ≥140 mmHg. Unfortunately, in both studies, neither version of olive oil changed diastolic blood pressure or arterial stiffness (Australian population).

Given the potent antioxidant and anti-inflammatory properties of polyphenolic compounds, Sarapis et al. (2023) demonstrated in a double-blind, crossover study (OLIVAUS) that consuming polyphenolic olive oil effectively reduces cholesterol peroxidation. After 3 weeks of consuming the appropriate olive oil variant, there was a significant decrease in oxLDL and a simultaneous increase in total antioxidant capacity in the group taking the high-polyphenol olive oil. Importantly, a decrease in C-reactive protein (CRP) of approximately 50% was observed in this group of patients. This is the first clinical study to confirm the correlation between the potential preventive activity of extra virgin olive oil polyphenols and risk factors in healthy individuals with early cardiovascular disease.

Promising results, although requiring further confirmation, were obtained by Sánchez-Quesada et al. (2020) regarding the effect of different types of olive oil on chronic arterial ischemia of the lower limbs (Ankle-Brachial Index). The PREDIMED-plus study (nearly 4,500 participants) showed that higher consumption of extra virgin olive oil was inversely associated with a low Ankle-Brachial Index, while consumption of pomace olive oil was positively associated with a low Ankle-Brachial Index.

Another randomized, placebo-controlled, crossover study assessed the effect of extra virgin olive oil (polyphenol concentration 161 mg/kg) and refined olive oil (polyphenol concentration 15 mg/kg) on inflammatory markers in patients with stable coronary artery disease. Fifty participants strictly followed the recommended diet and additionally consumed the appropriate olive oil for two 3-week periods, separated by 2-week washout periods using only low-polyphenol olive oil. Supplementation with high-polyphenol olive oil significantly reduced the secretion of interleukin-6 (IL-6) and CRP. Furthermore, increased concentrations of polyphenol metabolites such as tyrosol, hydroxytyrosol, and O-methylhydroxytyrosol were detected in urine (Fito et al., 2008). Using the same approach, Fito et al. (2005) demonstrated a decrease in plasma oxidized LDL and lipid peroxides, as well as a significantly increased glutathione peroxidase activity, after consuming extra virgin olive oil. However, neither study confirmed a correlation between extra virgin olive oil and a decrease in intercellular and vascular adhesion molecules, glucose concentration or changes in the lipid profile of patients (Fito et al., 2005; Fito et al., 2008).

### 5.2 Randomized controlled trials of purified Oleaceae metabolites

While there are a few clinical studies on various versions of olive oil, there are very few studies on individual active metabolites. On the one hand, the results of these studies confirmed that hydroxytyrosol has strong bioactivity in the prevention and treatment of cardiovascular diseases. However, on the other hand, no clinical studies were designed to assess the specific properties of hydroxytyrosol as a monocomponent. Domenech et al. (2019) only demonstrated that supplementation with a combination of nutraceuticals containing hydroxytyrosol (5 mg), monacolin K from red rice (10 mg), and phytosterols (1.5 g) significantly reduced cardioprotective parameters. Forty volunteers with elevated total and LDL cholesterol participated in a randomized, double-blind, placebo-controlled, parallel-group study. After 3 months, a significant decrease in the concentration of total cholesterol (approx. 12%), LDL fraction (approx. 19%) and apolipoprotein B (approx. 12.5%) as well as CRP (p = 0.021) in plasma was observed compared to the placebo group. In another randomized, double-blind, placebo-controlled, 20-week crossover study, Quirós-Fernández et al. (2019) evaluated the effect of oral supplementation with a preparation containing hydroxytyrosol (9.9 mg) and punicalagin (195 mg) on early markers of atherosclerosis in apparently healthy middle-aged adults. The preparation significantly reduced oxLDL by −28.74 ng/mL (p < 0.05) in those with higher oxLDL levels, which was an improvement compared with placebo (28.74 ± 40.2 vs. 25.64 ± 93.8 ng/mL, p < 0.001). An important aspect of this study was the decrease in blood pressure (systolic and diastolic, p < 0.001) in the groups with hypertension and prehypertension and the improvement of vascular endothelial function in the group with its dysfunction (p < 0.001).

Boronat et al. (2019) confirmed the bioconversion of tyrosol to hydroxytyrosol and assessed its recovery and its effect on certain cardiovascular parameters. A mixture of tyrosol (25 mg) and white wine, administered as capsules, was used in a randomized, crossover, controlled study (n = 33). The results confirmed the transformation of tyrosol to hydroxytyrosol. Furthermore, the recovery of hydroxytyrosol was statistically significant (p < 0.05). The mixture of tyrosol and white wine improved endothelial function, increased plasma HDL and antithrombin III levels, and reduced the expression of homocysteine, endothelin 1, and the CD40L, P65/RELA, and complement factor H (CFH) genes in peripheral blood mononuclear cells (p < 0.05). Moreover, capsules containing tyrosol and white wine inhibited the expression of iNOS, eNOS, VEGFA, and CHF stimulated by capsules containing only white wine (p < 0.05).

The remaining Oleaceae metabolites described in the article have not been the subject of clinical research, although they exhibit potent antioxidant and anti-inflammatory properties and regulate a number of signaling pathways responsible for the development of early and late atherosclerotic lesions. Some of these metabolites, such as syringin, are a key active ingredient in Traditional Chinese Medicine (TCM). Syringin is used in various TCM preparations due to its numerous pharmacological properties, including anti-inflammatory, hypolipidemic, and hypoglycemic properties. Syringin is known to have cardioprotective potential and is an ingredient in many drugs and medicinal products available in the Asian market, such as Aidi injections (Qian et al., 2024).

### 5.3 Potential side effects and interactions

Side effects and drug interactions are potential hazards resulting from the use of medicinal products. The risk of adverse reactions is particularly high in people with chronic diseases such as type 2 diabetes, hypertension, cancer, *etc.* Although natural ingredients are known to be relatively safe and side effects are less common after their use, they may also pose a potential risk to the patient. One rare article describes that oleuropein may exacerbate low blood pressure in individuals with hypotension. Furthermore, oleuropein may also interact with other antihypertensive and hypoglycemic medications (Sun et al., 2017a).

Most of the described Oleaceae metabolites are known to exhibit potent anti-inflammatory properties, including inhibition of the arachidonic acid pathway, which produces prostaglandin E2 (PGE2). This same signaling pathway leads to the synthesis of thromboxane A2. Therefore, taking hydroxytyrosol, oleuropein, oleacein, oleocanthal, acteoside, caffeic acid, chlorogenic acid, forsythoside A, oleanolic acid, phillygenin, syringin, and ursolic acid may increase the risk of bleeding. Moreover, these metabolites may interact with anticoagulants, as well as with some antidepressants and chemotherapy drugs. Oleocanthal has previously been described to have potent analgesic properties comparable to ibuprofen. This compound may interact with other nonsteroidal anti-inflammatory drugs.

Pharmacological interactions can also have positive effects. For example, oleocanthal may enhance the effects of anticancer drugs such as tamoxifen and lapatinib by interacting with their target proteins (Parkinson and Keast, 2014). Oleocanthal has also been shown to have the potential to enhance the effects of donepezil in Alzheimer’s disease (Batarseh and Kaddoumi, 2017). Similarly, oleacein can inhibit histone deacetylation enzymes (HDACs), leading to chromatin compaction and gene silencing. Oleacein, as an HDAC inhibitor, is being investigated for the treatment of certain types of cancer (Cuyàs et al., 2019).

### 5.4 Meta-analyses and regulatory health claims (EFSA, FDA)

The goal of meta-analysis in scientific research is to synthesize the results of multiple individual studies to obtain more reliable and general conclusions on a given topic. There are several studies confirming the beneficial effects of olive oil consumption on heart and vascular diseases. There are significantly fewer meta-analyses on individual metabolites of Oleaceae. Ussia et al. (2025) identified seventeen clinical studies that demonstrated an association between extra virgin olive oil consumption and a reduced risk of developing cardiovascular disease. In particular, improvements in biomarkers involved in cardiometabolic pathways and subsequent cardiovascular events were noted. This beneficial effect, supported by 20 years of research, was attributed to the polyphenolic compounds contained in olive oil. Martínez-Gonzalez et al. (2022) based on a systematic review and meta-analysis of prospective, randomized, and cohort studies, assessed the correlation between olive oil consumption and the risk of developing proinflammatory diseases, including atherosclerosis. Thirty-six studies were included in the analysis, allowing for the assessment of nearly 50,000 patients with cardiovascular disease. Furthermore, the frequency of olive oil consumption was measured using validated questionnaires. The authors of the meta-analysis found that for each additional 25 g serving of olive oil, there was a 16% decrease in the risk of cardiovascular disease (relative risk [RR]: 0.84; 95% confidence interval [CI]: 0.76–0.94). Interestingly, this study also observed an association between olive oil consumption and a reduced risk of type 2 diabetes, but not cancer. A meta-analysis (31 articles) of the health effects of olive oil consumption showed that olive oil significantly reduced the risk of cardiovascular disease and stroke. However, the results regarding coronary heart disease remained inconclusive. Furthermore, when evaluating the method of olive oil administration, it was found that liquid olive oil provided more benefits in lowering blood pressure than encapsulated olive oil (Chiavarini et al., 2024).

Tsartsou et al. (2019) assessed the role of individual groups of active compounds (polyphenols and MUFA) in olive oil on changes in metabolic factors (glucose, lipid profile, oxidants, proinflammatory factors). Thirty studies were selected for meta-analysis from approximately 1,000 studies, which considered direct and indirect interactions, as well as the impact of each component. The results of this study were surprising. They found that the effect of olive oil polyphenols on glucose levels (−0.105, 95% CI = −0.174, −0.036) and lipid profile (total cholesterol: *d* = −0.191, 95% CI = −0.259, −0.122, LDL: *d* = −0.189, 95%CI = −0.238, −0.140 and oxLDL: *d* = −0.112, 95% CI = −0.375, 0.150) depended on adherence to the Mediterranean diet. They also demonstrated that lower levels of bioactive polyphenols may be sufficient to maintain the therapeutic effect of olive oil, not only in terms of anti-inflammatory and antioxidant properties but also in increasing HDL levels (d = 0.113, 95% CI = 0.064, 0.163). On the other hand, it is important to remember that the therapeutic effect of polyphenols correlates with their concentration, as demonstrated in numerous studies using cellular and animal models. These results are consistent with a meta-analysis of clinical trials, which demonstrated that high-polyphenol olive oil is more effective in reducing CRP (39.4% and 35.86%) and proinflammatory cytokine concentrations (IL-6: 12.03%–33.3%; IL-7: 20.8%; IL-18: 2%–11.4%; IFNg: 9.25%) in blood serum (Fernandes et al., 2020; Tsartsou et al., 2019).

However, the term “olive oil polyphenols” is relative and encompasses a large group of compounds with varying bioactivity. In principle, only one meta-analysis exists for individual compounds, i.e., oleuropein and hydroxytyrosol, while the remaining metabolites of Oleaceae are still not well understood. Frumuzachi et al. (2025), based on 14 interventional studies (nearly 600 participants), assessed the effect of oleuropein and hydroxytyrosol supplementation on the risk of cardiometabolic diseases. The analysis showed that oleuropein and hydroxytyrosol significantly reduced the concentration of total cholesterol (SMD = −0.19, CI: 0.37 to −0.01, p = 0.04, I2 = 35%), triglycerides (SMD = −0.32, CI: 0.60 to −0.03, p = 0.03, I2 = 73%), and insulin (SMD = −0.42, CI: 0.82 to −0.01, p = 0.04, I2 = 78%). Importantly, supplementation with isolated compounds, not olive oil, was found to have a beneficial effect on the patients’ Body Mass Index (BMI). On the other hand, a previous meta-analysis showed no correlation between hydroxytyrosol and metabolic syndrome [combined standardized mean differences (SMD) = 0.01 (CI 95%: [−0.23, 0.25], I2 = 83%; p = 0.920)]. However, it was finally concluded that hydroxytyrosol supplementation showed a quite beneficial effect on antioxidant capacity related to metabolic syndrome components [SMD = 0.31 (CI 95%: [−0.34, 0.95], I2 = 81%]; p = 0.35) (Pastor et al., 2021).

Numerous *in vitro* and *in vivo* studies, as well as a small number of clinical trials, have contributed to the classification of olive oil, particularly polyphenol-rich olive oil, as a product with potential medicinal value. The European Food Safety Authority (EFSA) has confirmed that the polyphenols in olive oil help protect blood lipids from oxidative damage; therefore, extra virgin olive oil, a key component of the Mediterranean diet, can be a functional food ingredient. Such olive oil must contain at least 5 mg of hydroxytyrosol and its derivatives (oleuropein and tyrosol) per 20 g to qualify for an EFSA-approved health claim.

In turn, the U. S. Food and Drug Administration (FDA) has deemed hydroxytyrosol safe for use as a food ingredient with antioxidant and antimicrobial properties in certain foods, having been scientifically determined to be Generally Recognized As Safe. It's important to note that hydroxytyrosol can be used in beverages, fats and oils, fruits and vegetables, and sauces, at specified doses, but it is not approved for use in infant formula or meat products under USDA jurisdiction (GRAS Notice (GRN) No 600 for Hydroxytyrosol). Interestingly, however, no therapeutic treatments using hydroxytyrosol have received FDA approval.

### 5.5 Limitations and heterogeneity of existing trials

Due to the complex model and numerous variables, clinical trials are difficult to implement. Many promising projects fail because biological mechanisms fail to work in the complex conditions of the human body. Some studies involving olive oil alone have failed to confirm their hypotheses. For example, Sarapis et al. (2023) conducted a double-blind, randomized, crossover study (OLIVAUS) to assess the ability of high-polyphenol olive oil to enhance cholesterol efflux through the HDL fraction. Although *in vitro* studies confirmed the mechanism of cholesterol efflux for some biocomponents of extra virgin olive oil, i.e., oleacein (Filipek and Gierlikowska, 2021), this study did not demonstrate significant changes in HDL cholesterol efflux in either the low- or high-polyphenol olive oil treatment groups. Similarly, no significant association was found between metabolites from extra virgin (78 metabolites) and classic olive oil (74 metabolites) and the development of type 2 diabetes. The PREDIMED study enrolled nearly 2,000 patients at high cardiovascular risk. Although the results of extensive epidemiological studies indicated an association between key metabolite profiles (classic and extra virgin olive oil) and a reduced incidence of cardiovascular disease, this association did not extend to the Mediterranean population with type 2 diabetes (García-Gavilán et al., 2023). Moreover, too much variability in research designs, populations and types of olive oil did not allow for unambiguous confirmation of the effect of olive oil on anthropometric indicators (BMI) and inflammatory biomarkers, which indicates the need to systematize research protocols in future studies (Chiavarini et al., 2024).

A limitation of the use of the Mediterranean diet in clinical trials is the health-promoting effects of other biocomponents. It is important to remember that olive oil is rich in monounsaturated fatty acids (MUFA). Camargo et al. (2012) demonstrated that MUFA reduced the expression of genes associated with inflammation (e.g., NF-κB, MCP-1, TNF-α, and IL-6) and atherosclerotic plaque stability (e.g., MMP-9) in geriatric patients consuming a diet containing MUFA-enriched extra virgin olive oil.

It appears that a high-fat diet may have health consequences. Diets containing 3-4 tablespoons of olive oil are problematic due to their caloric content. This amount of olive oil provides 300–550 kcal, which covers 20%–25% of the daily requirement of an average woman and 17%–20% of the average man. Obesity is a global health problem, affecting over a billion adults and many adolescents worldwide. It is estimated that by 2035, over half the world’s population could be overweight or obese. It is also known that excess body fat regulates the functioning of the entire body and leads to the development of pro-inflammatory diseases such as hypertension, atherosclerosis, thrombosis, hypercholesterolemia, and type 2 diabetes.

Clinical trial heterogeneity refers to the heterogeneity in patients, treatments, interventions, or outcomes across studies that are compared or integrated within a meta-analysis. This heterogeneity can include patient characteristics such as age (geriatric, young), gender, disease stage (healthy individuals, those with stable coronary artery disease, those with hypertension, those with type 2 diabetes, those with prediabetes, *etc.*), study methodology (protocols, doses of biocomponents, *etc.*), or outcome measurement, which makes it difficult to combine results and draw common conclusions. Therefore, although the observational studies and meta-analyses discussed in this article indicate an association between high olive oil consumption and a lower risk of mortality, particularly from cardiovascular disease, larger, multicenter, randomized, controlled trials are needed to confirm these findings and better understand the specific mechanisms.

## 6 Discussion

Bioactive metabolites derived from Oleaceae plants including secoiridoids, phenylethanoids, phenylpropanoids, triterpenoids and lignans exhibit substantial antioxidant, anti-inflammatory, lipid-modulating and endothelial-protective effects across *in vitro* and *in vivo* models of atherosclerosis. Collectively, the preclinical evidence supports their ability to modulate multiple pathways relevant to plaque initiation, progression and destabilization, including NF-κB inhibition, Nrf2/HO-1 activation, ABCA1/ABCG1 – mediated cholesterol efflux, suppression of adhesion molecules, inhibition of CD36, attenuation of ROS generation and stabilization of extracellular matrix components. In animal models, key metabolites such as hydroxytyrosol, oleuropein, oleacein, oleanolic acid, ursolic acid, caffeic acid and chlorogenic acid consistently reduce plaque burden and ameliorate systemic markers of inflammation and dyslipidemia.

Clinical data, while comparatively limited, largely support these findings. Human studies, primarily involving extra virgin olive oil (EVOO), demonstrate improvements in endothelial function, reductions in oxidized LDL and inflammatory markers, favorable modulation of lipid profiles and, in large cohorts (e.g., PREDIMED), reduced incidence of cardiovascular events. However, most human trials assess complex dietary substances rather than purified phytochemicals, and the polyphenolic composition of EVOO varies significantly between cultivars and processing methods.

### 6.1 Limitations of the evidence and potential publication bias

Despite promising results, several limitations should be noted. Not all animal studies are randomized, blinded, or dosed in a standardized manner. Furthermore, most experiments are conducted in young male rodents, limiting generalizability. *In vitro* and *in vivo* studies frequently use concentrations that exceed physiologically achievable levels. Moreover, publication bias is likely, as studies with positive antioxidant or anti-inflammatory results are more commonly reported in phytochemical research. Negative results are rarely reported, contributing to an overestimation of therapeutic potential.

### 6.2 Safety considerations, dosing variability and differences between purified metabolites from Oleaceae plants and dietary sources

Safety data for isolated metabolites remain scarce. Most clinical trials evaluate extra virgin olive oil rather than isolated secoiridoids or triterpenoids. The polyphenol content of olive oil differs substantially by cultivar, processing method, and storage conditions, which complicates dose-response interpretation. Consequently, clinical dosing across studies is inconsistent and difficult to compare. Purified metabolites, particularly hydroxytyrosol, oleuropein and oleocanthal may reach higher plasma concentrations but may also pose risks for drug-supplement interactions, especially through CYP450 modulation and antiplatelet activity. Long-term safety of high-dose purified metabolites from Oleaceae plants has not been established.

### 6.3 Bioavailability challenges and translational barriers

A significant barrier to clinical translation is poor oral bioavailability. Triterpenoids (oleanolic and ursolic acids) exhibit low water solubility and extensive first-pass metabolism. Secoiridoids (oleuropein and oleacein) undergo extensive hydrolysis and phase-II conjugation. Metabolites, such as hydroxytyrosol sulfate and glucuronide conjugates, may contribute more to biological activity than parent molecules. These issues create uncertainty regarding the doses required to reproduce effects observed in animal models. Most mouse studies use doses equivalent to hundreds of milligrams per day in humans, often well above what can be realistically achieved through diet. For example, a typical human olive oil intake provides ∼5–10 mg hydroxytyrosol equivalents per day, far lower than doses used in rodent studies (10 mg/kg). Achieving therapeutic plasma levels may require concentrated nutraceutical formulations, improved delivery systems (e.g., lipid nanoparticles), or structural derivatives with enhanced absorption.

### 6.4 Feasibility, regulatory considerations and future perspectives

Few Oleaceae-derived metabolites have regulatory approval as therapeutic agents. They are mostly marketed as dietary supplements, where purity, standardization, and stability vary considerably. Any future translation would require: standardized manufacturing of purified compounds; rigorous toxicity and pharmacokinetic studies; assessment of drug interactions, especially with statins, anticoagulants, and antiplatelet agents; well-controlled clinical trials using quantified doses.

## 7 Conclusion

In summary, plants belonging to the Oleaceae family are an excellent source of anti-atherosclerotic metabolites. Various *in vitro* and *in vivo* studies have demonstrated their multi-targeted activity in both the early and late stages of atherosclerosis, but their clinical application remains limited. Future work should therefore focus on conducting adequate and well-controlled clinical trials with defined doses, validated biomarkers and clear cardiovascular endpoints. Integrating these metabolites into preventive and adjunctive therapeutic strategies may offer a safe, multi-targeted approach to atherosclerosis management, but this requires further research.
