# Supplementary material for: Oleaceae plants: a source of metabolites with atheroprotective potential
Source: Front Pharmacol. 2026 Feb 11;16:1721433. doi: 10.3389/fphar.2025.1721433 (PMC12932613; doi:10.3389/fphar.2025.1721433)
Supplement: Supplementary file 1 [file DataSheet1.pdf]

Suplemmentary materials

No	Metabolites	Source of metabolites		Identity confirmation plant materials	Isolation methods	Identity confirmation of metabolites	Purity	Literature
		Commercial	Plant materials					
1.	Hydroxytyrosol	Cayman Chemical	-	-	-	-	≥98%	Bender et al., 2023<br>Zhang et al., 2020
		Extrasyntese (Genay, France)	-	-	-	-	>99%	Castejon et al., 2022
		TCI (Zwijndrecht, Belgium)	-	-	-	-	≥98%	Cuffaro et al., 2024
		Sigma-Aldrich (St. Louis, MO, USA)	-	-	-	-	≥98%	Del Saz-Lara et al., 2025<br>Miao, Geng, Ning, 2023<br>Zodio et al., 2024
		Natac, Madrid, Spain	-	-	-	-	>98%	Franceschelli et al., 2023
		TCI (Belgium).	-	-	-	-	>98%	Gabbia et al., 2023
		Tokyo Chemical Industry Co.,	-	-	-	-	>98%	Yonezawa et al., 2018

		Ltd., Tokyo, Japan						
		Viablife Biotech Co (Hangzhou, China)	-	-	-	-	99%	Wen et al., 2024
		-	Extra virgin olive oil (2019–2020 harvesting period)	-	FCPE<br>FCPC<br>TLC	LC-HRMS<br>NMR<br>HPLC-DAD	98.5%	Christodoulou et al., 2024
		-	Samples of alperujo, a very wet solid waste from two-phase decanters, were supplied by the oil extraction factory Oleícola El Tejar (Córdoba, Spain). Alperujo was sampled at three different dates in the olive oil production season. The first sample was taken on November 25, at the beginning of the olive oil production season; the	-	HPLC	HPLC/UV<br>NMR	>95%	Montoya et al., 2018

			second sample was taken on January 28, at the halfway mark of the season, and the third sample was taken on March 26, at the end of the season.					
		Unilever Research & Development Vlaardingen, The Netherlands.	extra-virgin olive oil	-	HPLC	HPLC-MS-MS	-	Leenen et al., 2002
		N-Zyme BioTec GmbH (Darmstadt, Niemcy)	-	-	FCPC	chemical standards	98%	Grewal et al., 2020
		Tokyo Chemical Industry Co., Ltd.	-	-	-	-	≥98%	Hara et al., 2023
2.	Oleuropein	Cayman Chemical	-	-	-	-	≥98%	Bender et al., 2023
		Extrasyntese (Genay, France)	-	-	-	-	>99%	Castejon et al., 2022

		Vinci-Biochem Srl (Florence, Italy)	-	-	-	-	≥98%	Delle Monache et al., 2021
		Indofine Chemical Company, Inc. (Belle Mead, NJ)	-	-	-	-	>98%	Edgecombe et al., 2000
		N-Zyme BioTec GmbH (Darmstadt, Niemcy)	-	-	FCPC<br>HPLC	chemical standards	96%	Grewal et al., 2020
		Sigma-Aldrich (St. Louis, MO, USA	-	-	-	-	>98%	Hu et al., 2025<br>Wang et al., 2017
		-	Olea europea var. Koronoiki, Oleaceae, Olea europaea folium	Koronoiki	FCPE<br>FCPC<br>TLC	LC-HRMS<br>NMR<br>HPLC-DAD	98%	Christodoulou et al., 2024

		-	*Ligustrum vulgare* L., Oleaceae, *Ligustrum vulgare folium*	Ligustrum vulgare leaves was deposited in the plant collection at the Department of Pharmacognosy and Molecular Basis of Phytotherapy, Medical University of Warsaw (No LV062012). Ligustrum vulgare leaves (52°25′11.272″N, 20°55′15.277″E) were dried at room temperature with no exposure to light.	HPTLC, UHPLC-DAD-MS	HPLC-DAD, NMR	>95%	Kiss, Mank, Melzig, 2008 Parzonko et al., 2013
		-	derived from powdered olive leaf extract;	-	-	-	-	Mirsanei et al., 2023

3.	Oleacein	-	*Ligustrum vulgare* L., Oleaceae, *Ligustrum vulgare folium*	Ligustrum vulgare leaves was deposited in the plant collection at the Department of Pharmacognosy and Molecular Basis of Phytotherapy, Medical University of Warsaw (No LV062012). Ligustrum vulgare leaves (52°25′11.272″N, 20°55′15.277″E) were dried at room temperature with no exposure to light.	HPTLC, UHPLC-DAD-MS	HPLC-DAD, NMR	>95%	Kiss, Mank, Melzig, 2008<br>Filipek et al., 2017<br>Filipek et al. 2021<br>Parzonko et al., 2013

		-	*Olea europaea* L. Oleaceae, *Olea europaea folium*	Olive leaves were collected in November from cultivar Leccino in the Marche region (Italy).	UHPLC-UV-ESI-HRMS	NMR		Silvestrini et al., 2023
		-	Olive oil from Koroneiki (northern Peloponnese, Greece)	-	HPTLC H-NMR	qNMR	>95%	Gutierrez-Miranda et al., 2020
		-	Three EVOOs were selected: two Tuscan EVOOs of the 2019/2020 crop seasons (A and B) and an Italian EVOO of the	-	HPLC	HPLC-UV chemical standars	-	Salsano et al., 2022

			2018/2019 crop season					
		N-Zyme BioTec GmbH (Darmstadt, Niemcy)	-	-	HPLC	HPLC-MS NMR	96%	Grewal et al., 2020
		Toronto Research Chemicals (North York, ON, Canada)	-	-	-	-	>90%	López-Yerena et al., 2021
4.	Oleocanthal	Sigma-Aldrich	-	-	-	-	≥95%	Banaei et al., 2025
		N-Zyme BioTec GmbH (Darmstadt, Niemcy)	-	-	FCPC	HPLC-MS NMR	96%	Grewal et al., 2020
		-	Ligustrum vulgare L. Oleaceae, *Ligustrum vulgare folium*	1. *Ligustrum* leave s were collected in 2022 and 2023 from Bamboo Brook Outdoor Recreation Center and Willowwood Arboretum, Northern New Jersey (USA). 2. Leaves from *L. vulgare* (or common privet) and *L. ibolium* (or	UHPLC	LC-MS NMR	-	Peyrot des Gachons et al., 2024

				North privet) were collected on 27 September 2022, from six different 2-year-old plants of each cultivar, which were all planted in an isolated section of Willowwood arboretum.<br>3. Leaves from *L. vulgare* and *L. obtusifolium* were collected at Bamboo Brook and those of *L. ibolium* at Willowwood. *L. ovalifolium* plants were obtained from Bay Gardens Nursery in East Moriches, New York, and *Olea europeae* Koronei ki (olive) plants came from Brighter Blooms Nursery in South Carolina.				

		-	Extra virgin olive oil (2019–2020 harvesting period)	-	FCPE<br>FCPC<br>TLC	LC-HRMS<br>NMR<br>HPLC-DAD	98.5%	Christodoulou et al., 2024
		-	Greek extra virgin olive oil “Kloster Toplou” purchased from Hofer KG, Sattledt, Austria.	-	HPCCC	LC–MS<br>^{13}C NMR	-	Wang et al., 2017
		-	extra virgin olive oil, Arbequina cultivar	-	HPLC	HPLC<br>^{1}H NMR<br>^{13}C NMR	>95%	Montoya et al., 2019
		-	Extra virgin olive oil Cornicabra cultivar	-	HPLC	HPLC<br>^{1}H NMR<br>^{13}C NMR	>95%	Montoya et al., 2021<br>Montoya et al., 2023

			EVOO were used (OO500, OO250, and OO) (23); the OO500 was produced by Kalamon olives variety (Olea europaea L.), which was harvested in November. The malaxation was for 45 min at 30°C, using the OMPHAX SA olive mill's facilities			qNMR		Katsa et al., 2024
		-	Extra virgin olive oil EVOO; Member's Mark, batch no. VF1-US102808, Italy	-	HPLC<br>TLC	HPLC<br>^{1}H NMR	>90%	Qosa et al., 2015
5.	Oleanolic acid	-	Extra virgin olive oil (2019–2020 harvesting period)	-	FCPE<br>FCPC<br>TLC	LC-HRMS<br>NMR<br>HPLC-DAD	98.5	Christodoulou et al., 2024
		Solarbio (Beijing, China)	-	-	-	-	≥98%	Dong et al., 2020<br>Zhang et al. 2018

		Sigma-Aldrich (St. Louis, MO, USA)	-	-	-	-	≥98%	Hwan et al., 2014<br>Wang et al., 2017
		Nianjing Jiancheng (Nianjing, China)	-	-	-	-	>98%	Jiang et al., 2015
		Santa Cruz, Dallas, USA	-	-	-	-	>98%	Peng et al., 2019
		Selleck, United States	-	-	-	-	-	Li et al., 2021
		Extrasynthese (Genay, France)	-	-	-	-	≥99%	Marquez-Martin et al. 2006<br>Martin et al., 2014
		MCE, China	-	-	-	-	98%	Wang et al., 2024
		(Merck, Darmstadt, Germany	-	-	-	-	>97%	Stelling-Ferez et al., 2023
		NIEHS, Research Triangle Park, NC	-	-	-	-	-	Reisman, Aleksunes, Klaassen, 2009

		-	*Forsythiae viridissima* Lindl.*, Oleaceae Forsythiae viridissimafructus*	Forsythiae Fructus, *Forsythiae viridissima* fruits were collected near Sokri Mountain in the Republic of Korea.	HPLC-UV	Chemical Standards	>95%	Lee et al., 2010
6.	Ursolic acid	Sigma-Aldrich (St. Louis, MO, USA)	-	-	-	-	≥98%	Hwan et al., 2014<br>Jiang et al., 2016<br>Ma, Ding, Zhang, Liu, 2015<br>Wang et al., 2017<br>Yu et al., 2017<br>Zhang et al., 2017
		Royan Company	-	-	-	-	>98%	Pordanjani, Banitalebi,Roghani, Hemmati, 2022
		Aladdin (Beijing, China)	-	-	-	-	>98.5%	Ma et al., 2022
		Chengdu Herbpurify Co. Ltd. (Chendu, China)	-	-	-	-	≥98%	Li et al., 2018
		-	*Forsythiae viridissima* Lindl.*, Oleaceae*	Forsythiae Fructus, *Forsythiae viridissima* fruits were collected	HPLC-UV	Chemical Standards	>95%	Lee et al., 2010

			Forsythiae viridissimafructus	near Sokri Mountain in the Republic of Korea.				
7.	Erythrodiol	Extrasynthese (Genay, France)	-	-	-	-	≥97%	Marquez-Martin et al. 2006
		Sigma (St. Louis, MO, USA)	-	-	-	-	>95%	Penas-Fuentes et al., 2021<br>Wang et al., 2017
8.	Forsythoside A	Chengdu Herbpurify Co., Ltd. (Chengdu, China)	-	-	-	-	≥98%	Cheng et al., 2013
		MUST Biotechnology Co., Ltd. (Chengdu, China)	-	-	-	-	>98%	Fu et al., 2023
		MCE, Shanghai, China	-	-	-	-	>99%	Li et al., 2025
	Forsythoside B	Shanghai Yuanye Biotechnology Co., Ltd. (China)	-	-	-	-	>98%	Chen et al., 2023

		MedChemExpress, Weehawken, USA)	-	-	-	-	99.9%	Xia et al., 2022
		Guangdong Provincial Key Laboratory of New Drug Development and Research of Chinese Medicine, Guangzhou University of Chinese Medicine (Guangzhou, China)	-	-	-	-	>98%	Zheng et al., 2021
9.	Syringin	Yuan Ye Bio-Technology (Shanghai, China)	-	-	-	-	≥98%	Cong et al., 2025
		MedChemExpress, China					>98%	Zhao et al., 2023
		Rainbow Biotechnology Co. Ltd. (Shilin, Taipei, Taiwan)	-	-	-	-	98%	Fu et al., 2022
		-	*Syringa vulgaris* L., Oleaceae, *Syringa vulgaris cortex*	Bark from the branches of *Syringa vulgaris* L. was collected in May	TLC HPLC-DAD-MS/MS	HPLC-DAD-MS/MS NMR	>95%	Filipek et al., 2019

				2016 from a native state in Warsaw, Mazovian district, Poland (52°15′56.0″N 20°59′35.1″E). The plant material was authenticated according to Flora Europaea by Anna K. Kiss. A voucher sample (no. SV052017) was deposited in the Plant Collection, Department of Pharmacognosy and Molecular Basis of Phytotherapy, Medical University of Warsaw, Poland				

		-	*Tinospora crispa* L. Menispermaceae , *Tinospora crispa caulis*	A voucher specimen (UKMB 40178) was identi-fied by Dr. Abdul Latif Mohamad of Faculty of Science and Technology, Universiti Kebangsaan Malaysia (UKM) and deposited at the Herbarium of UKM, Bangi, Malaysia.	HPLC, LOQ, LOD	LC-MS-MS, NMR	>95%	Arshad et al, 2024
		-	*Rhaponticum carthamoides (Willd., Asteraceae, Rhaponticum carthamoides herba*	The dried medicinal herbs of RC (Batch No. 20170622) were purchased from Xingshengtai Biotechnology Co., Ltd. (Xinjiang, China) and stored at the School of Pharmacy of Xi'an Jiaotong University	HPLC	HPLC Chemical Standards	-	Nanet al., 2024

9.	Phillygenin	Chengdu Must Biochemical Technology Co., Ltd. (Chengdu, China)	-	-	-	-	>98%	Guo et al., 2022<br>Ma et al., 2023<br>Zhou et al., 2021
		-	*Forsythia x intermedia* Zabel, Oleaceae, *Forsythia x intermedia flos*	The flowers and leaves of Forsythia x intermedia Zabel were collected in April and June 2016, respectively, in the Arboretum, Forestry Experimental Station of Warsaw University of Life Sciences in Rogow, Poland (51°49′ N; 19°53′E). The identity of the plant material was confirmed by Piotr Banaszczak, Head of the Arboretum, Forestry Experimental Station of Warsaw University of Life Sciences. A voucher specimen	HPLC-DAD-MS/MS	UHPLC-DAD-MSn<br>NMR	>95%	Michalak et al., 2018

				(FINT-F-2013) has been deposited in the herbarium of the Department of Pharmacognosy, Medical University of Lodz, Poland.				
		-	Osmanthus fragrans Loureiro, Oleaceae, Osmanthus fragrans *flos*	Osmanthus fragrans was purchased from a traditional market at Guilin, Guangxi Province, China, in 2007. Voucher specimen of *Osmanthus fragrans* (HCY080801) has been deposited at the herbarium of the Department of Food Nutrition, Chung Hwa University of Medical Technology, Tainan, Taiwan.	HPLC, LOQ, LOD	NMR<br><br>^{13}C NMR	>95%<br><br>>98,6%	Hung et al., 2012<br><br>Song et al., 2018
		-	*Forsythia suspensa* L., Oleaceae,	F. suspensa dried fruits were purchased from an	-	-	-	Sung et al., 2016

			Forsythia suspensa fructus	oriental drug store (Omniherb Co., Yeoungcheon, Korea). A voucher specimen (No. KIOM-78039) was deposited at the herbarium of the Department of Herbal Resources Research of KIOM (Daejeon, Korea).				

		-	*Forsythia suspensa* L., Oleaceae, *Forsythia suspensa folium*	*Forsythia suspensa* leaves were collected in May 2018 in Arboretum, Shanxi University, Shanxi Province of China. After being dried in the shade, the plant was fermented to extract phillygenin, and part of the dried plant samples were stored in the Institute of Molecular Science of Shanxi University for future reference (No. 18070702).	HPLC, LOQ, LOD	UPLC	96%	Guo et al., 2022

			From natural products was purchased from the National Institute for the Control of Pharmaceutical and Biological Products (Beijing, China)	-	-	HPLC	>98%	Liu et al., 2009
10.	Acteoside	Equl Corporation (Shanghai, China	-	-	-	-	≥98%	Chen et al., 2009
		V4015, Merck Ltd., Beijing, China	-	-	-	-	99%	Fan & Zhang, 2021
		Chengdu Must Bio-technology Co., Ltd. (Sichuan, China)	-	-	-	-	>98%	Jia et al., 2023
		Sigma-Aldrich (Steinheim, Germany)	-	-	-	-	>98%	Yang et al., 2023
		Shanghai Yuanye Bio-Technology Co., Ltd (Shanghai, China)	-	-	-	-	≥ 98%	Li et al., 2023<br>Wang et al., 2021

		-	*Cistanche tubulosa* (Schenk), Wight Orobanchaceae	The Cistanche tubulosa was purchased in November 2023 from Moyu County in the Hotan region of Xinjiang. It was identified as Cistanche tubulosa (Cistanche tubulosa (Schenk) Wight by Professor Haibaier, a researcher at Xinjiang Hetian College. The sample (Nos. GN01001) is deposited at the Institute of Agricultural Products Processing and Storage, Xinjiang Academy of Agricultural Sciences	HPLC-DAD	HPLC	>95%	Muhtar et al., 2025
		-	*Cistanche deserticola* Ma., Orobanchaceae	The dried slices of the succulent stem of C. deserticola (8.5% moisture content) were purchased from	HPLC-UV-Vis	QTOF-MS/MS	>95%	Jia et al., 2023

				Kangmei Traditional Chinese Medicine City (Bozhou, Anhui, China) and authenticated by an associate professor of pharmacy Zhang Limu of Tai'an First People's Hospital.				
11.	Ligstroside	Extrasyntese (Genay, France)	-	-	-	-	-	Castejon et al., 2022
		N-Zyme BioTec GmbH (Darmstadt, Niemcy)	-	-	FCPC	HPLC-MS NMR	96%	Grewal et al., 2020
12.	Oleoacteoside	-	*Fraxinus excelsior L.*, Oleaceae, *Fraxinus excelsior folium*	Leaves of *Fraxinus excelsior* were collected in June and July 2017 from native plants growing in Warsaw, Mazovian district (52°12′42″ N 21°00′07″ E), Poland. The plant materials were authenticated according to Flora Europaea by Anna	HPLC-DAD-MS/MS TLC	HPLC-DAD-MSn NMR	>95%	Kołtun-Jasion et al., 2023

				K. Kiss. A voucher specimen (no. FE062017) was deposited in the Plant Collection, Department of Pharmacognosy and Molecular Basis of Phytotherapy, Medical University of Warsaw, Poland.				
13.	Chlorogenic acid	Shanghai Yuanye Biological Technology Co., Ltd., Shang- hai, China)	-	-	-	-	≥98%	Dai et al., 2024
		Sigma-Aldrich (Steinheim, Germany and St. Louis, MO, USA)	-	-	-	-	≥98%	Geana et al., 2023<br>Li et al., 2022<br>Yuan et al., 2017
		Sigma-Aldrich, Cat. No. C3878, St. Louis, MO, USA	-	-	-	-	≥95%	Ong et al., 2013<br>Hada et al., 2020<br>Sato et al., 2011<br>Kim et al., 2024
		Macklin Company	-	-	-	-	>98%	Xue et al., 2024

		(Shanghai, China)						
		National Institutes for Food and Drug Control (Beijing, China)	-	-	-	-	≥98%	Wu et al., 2014
			Ipomoea batatas L., Convolvulacea, *Ipomoea batatas folium*	Food Science Institute, Zhejiang Academy of Agricultural Sciences.	-	-	-	Gu et al., 2023
14.	Caffeic acid	Chengdu Must Biotechnology Co., Ltd. (Chengdu, China)	-	-	-	-	≥98%	Cao et al., 2019
		Adamas Reagent (Shanghai, China)	-	-	-	-	≥98%	Cen et al., 2025
		TCI (Zwijndrecht, Belgium)	-	-	-	-	≥98%	Cuffaro et al., 2024
		Extrasynthese (Genay, France	-	-	-	-	>99%	Zielińska et al., 2021

		Sigma-Aldrich (Steinheim, Germany and St. Louis, MO, USA)	-	-	-	-	≥98%	Geana et al., 2023<br>Hwan et al., 2014<br>Sato et al., 2011<br>Khan et al., 2012<br>Kim et al., 2024<br>Sun et al., 2023<br>Wan et al., 2021<br>Zhang et al., 2025a<br>Zhang et al., 2025b
15.	Hydroxytyrosol +white wine (capsule)	-	-	-	-	LC-MSMS (1.3 ± 0.1 mg/L)		Boronat et al., 2019
16.	EVOO extracts	-	Extra virgin olive oil	two different Mediterranean cultivars (A: Frantoio and B: Leccino). The samples, produced by a three-phase oil mill, were collected in 2022 at two different times of crushing season: in early-stage October 2022 (A1 and B1)	-	HPLC<br>HPLC-MS-MS<br>FCR	-	Cuffaro et al., 2024

				and in later-stage November 2022				
17.	OMWW extract	-	Oil mill	two different Mediterranean cultivars (A: Frantoio and B: Leccino). The samples, produced by a three-phase oil mill, were collected in 2022 at two different times of crushing season: in early-stage October 2022 (A1 and B1) and in later-stage November 2022		HPLC<br>HPLC-MS-MS<br>FCR		Cuffaro et al., 2024
		OLIVEX HT6® (GROUPE GRAP'SUD)	-	-	-	Polyphenols 30%<br>Hydroxytyrosol 6%	-	Hara et al., 2023
18.	Aquilea Colesterol® Novel Nutraceutic	-	-	-	-	5mg hydroxytyrosol /sachet	-	Domenech et al., 2019
19.	VOO (virgin olive oil), COO (rafined olive oil)	-	-	-	-	Spanish food composition tables	-	García-Gavilán et al., 2023
20.	EVOO (extra virgin olive oil) VOO (virgin olive oil)	-	-	-	UHPLC-HRMS	Phenolic compounds UHPLC-HRMS	-	Geana et al., 2023

	VOOt (virgin olive oil obtained from olive cakes)							
21	Olive oil	-	Olea europaea L Oleaceae, Olea eropaea fructus	Olives (*Olea europaea* L. cv. Picual) were hand-harvested from rainfed orchards in Jaén, southern Spain, during the 2023–2024 crop season. The maturity index of the olives was 1.4	Phenolic compouds<br><br>Volatile Compounds HS-SPME	Phenolic compouds HPLC, references<br><br>Volatile Compounds GC-FID	-	Peralta, Vidal, Espinola, Ocana, Moya, 2025
22	VOO	San Francisco de Asís Cooperative (Montefrío (Granada), Spain)	-	-	-	-	-	Sanchez-Rodriguez et al., 2018
23	ROO	-	refined olive oil	ROO and VOO, came from the same cultivar and harvest and were prepared for the	-	Phenolic compounds HPLC	-	Fito et al., 2005

24	VOO	-	Virgin olive oil	present clinical trial<br><br>ROO and VOO were provided by the Olive Oil Coop- erative Association of Catalonia	-	Phenolic compounds HPLC	-	Fito et al., 2005
25	FVOO	-	Fuctional virgin olive oil	-	UPLC–ESI-MS/MS	hydroxytyrosol	-	Valls et al., 2015
26	VOO	-	Standard olive oil	-	UPLC–ESI-MS/MS	hydroxytyrosol	-	Valls et al., 2015
27	Supplement capsule	Mediteanox®, Euromed S.A., Barcelona, Spain	-	-	-	hydroxytyrosol 3,3 mg	-	Quirós-Fernández et al., 2019
28	Suplement capsule	Biosearch S.A. (Granada, Spain)	-	-	-	oleuropein-enriched (15% w/w) olive leaf extract (OLE)	-	Romero et al., 2016
29	Oleacein synthesis	The starting material for the synthesis of (–)-oleacein [(–)-oleuropein] was purchased from	-	-	FCPC	NMR	-	Rosilla et al., 2024

		Sigma-Aldrich (Madrid, Spain)						
30	Oleacein synthesis	Oleae europaea was commercially purchased from SHIN-SEI Co., Ltd.	Synthesis from oleuropein. Oleeuropein isoleted from Olea eropaea L, Oleaceae, Olea eropaea folium	-	ESI–MS	NMR	-	Shimamoto et al., 2023
